# Generalizing age effects on brain structure and cognition: A two‐study comparison approach

**DOI:** 10.1002/hbm.24524

**Published:** 2019-01-22

**Authors:** Christiane Jockwitz, Susan Mérillat, Franziskus Liem, Jessica Oschwald, Katrin Amunts, Svenja Caspers, Lutz Jäncke

**Affiliations:** ^1^ Institute of Neuroscience and Medicine (INM‐1) Research Centre Jülich Jülich Germany; ^2^ Department of Psychiatry, Psychotherapy and Psychosomatics Medical Faculty, RWTH Aachen University Aachen Germany; ^3^ University Research Priority Program Dynamics of Healthy Aging University of Zurich Zurich Switzerland; ^4^ JARA‐BRAIN, Jülich‐Aachen Research Alliance Jülich Germany; ^5^ C. & O. Vogt Institute for Brain Research Medical Faculty, Heinrich Heine University Düsseldorf Germany; ^6^ Institute for Anatomy I Medical Faculty, Heinrich Heine University Düsseldorf Germany; ^7^ Division of Neuropsychology University of Zurich Zurich Switzerland

**Keywords:** aging, brain structure, cognition

## Abstract

Normal aging is accompanied by an interindividually variable decline in cognitive abilities and brain structure. This variability, in combination with methodical differences and differences in sample characteristics across studies, pose a major challenge for generalizability of results from different studies. Therefore, the current study aimed at cross‐validating age‐related differences in cognitive abilities and brain structure (measured using cortical thickness [CT]) in two large independent samples, each consisting of 228 healthy older adults aged between 65 and 85 years: the Longitudinal Healthy Aging Brain (LHAB) database (University of Zurich, Switzerland) and the 1000BRAINS (Research Centre Jülich, Germany). Participants from LHAB showed significantly higher education, physical well‐being, and cognitive abilities (processing speed, concept shifting, reasoning, semantic verbal fluency, and vocabulary). In contrast, CT values were larger for participants of 1000BRAINS. Though, both samples showed highly similar age‐related differences in both, cognitive abilities and CT. These effects were in accordance with functional aging theories, for example, posterior to anterior shift in aging as was shown for the default mode network. Thus, the current two‐study approach provides evidence that independently on heterogeneous metrics of brain structure or cognition across studies, age‐related effects on cognitive ability and brain structure can be generalized over different samples, assuming the same methodology is used.

## 1 INTRODUCTION

As we get older, our brain undergoes substantial structural changes that seem to be related to changes in behavior (i.e., cognitive decline in older adults). However, previous research has shown that it is far from simple to bring the two domains—namely brain structure and behavior—together (Fjell et al., 2006; Jockwitz et al., 2017; Liu et al., 2011; Raz & Rodrigue, 2006; Ziegler, Dahnke, Gaser, & Alzheimer's Disease Neuroimaging, 2012). One important reason for this is that age‐related changes in both domains are complex and insufficiently understood. For example, large between‐study heterogeneity of designs and methods, differences in sample characteristics and the generally larger interindividual variability in samples of older adults hamper the extraction of consistent findings regarding age‐related changes in brain structure in the existing literature.

Still, what we can conclude from previous work so far is that effects of age are not homogeneous across the brain, but depend on (a) the functional properties of the brain region of interest (e.g., association cortices vs. primary sensory cortices), (b) the brain tissue (e.g., gray and white matter), (c) the brain structure metric looked at (e.g., brain volume‐based vs. surface‐based metrics or cortical thickness [CT] vs. surface area), and (d) methodological choices made during processing and analyses (e.g., differences in spatial smoothing) (Dickie et al., 2013; Fjell et al., 2014; Fjell, McEvoy, et al., 2014; Hogstrom, Westlye, Walhovd, & Fjell, 2013; Liem et al., 2015; O'Sullivan et al., 2001; Salat et al., 2005; Sowell et al., 2003; Walhovd et al., 2011; Ziegler, Dahnke, Jancke, et al., 2012).

Although there is a more solid database when it comes to cognitive aging (Schaie (1993); Schaie and Willis (2010); Schaie, Willis, and Caskie (2004); for reviews, see Harada, Love, and Triebel (2013); Kaup, Mirzakhanian, Jeste, and Eyler (2011); Salthouse (2010) it has also been established that—in analogy to brain aging—age‐related changes in cognitive abilities are complex. First, different cognitive abilities are differentially sensitive to age effects. Abilities such as processing speed, executive functions, episodic, and working memory have shown to be more vulnerable to age‐related decline as compared to verbal memory and world knowledge (Habib, Nyberg, & Nilsson, 2007; Hedden & Gabrieli, 2004; Park & Reuter‐Lorenz, 2009; Schaie et al., 2004; Schaie & Willis, 2010). And second, several studies suggest that cognitive performance follows nonlinear trends from early to late adulthood with a higher interindividual variability in older adults (Habib et al., 2007; Hartshorne & Germine, 2015; Hedden & Gabrieli, 2004). Hence, it is difficult to generalize results from one sample to another and, therefore, to draw reliable conclusions. Considering, for example, that lifespan trajectories of structural atrophy vary between brain regions (Fjell et al., 2013; Hogstrom et al., 2013; Sowell et al., 2003; Walhovd et al., 2011; Ziegler, Dahnke, Jancke, et al., 2012), age‐related differences in brain atrophy might not be replicable across samples when they do not match with respect to age distributions or other sample characteristics.

At this time, there is a clear progress toward brain imaging consortia and multicenter studies, such as ENIGMA (Thompson et al., 2014), the German National Cohort study (Nationale Kohorte; NAKO (Bamberg et al., 2015; German National Cohort, 2014), ADNI (Alzheimer's Disease Neuroimaging Initiative; Jack Jr. et al., 2008), U.K. Biobank (Miller et al., 2016; Sudlow et al., 2015), or Lifebrain (Walhovd et al., 2018). In the field of healthy aging, such projects use data pooling procedures (i.e., joint analysis of data from different independent samples) to fulfill the need for large sample sizes required to identify protective and risk factors that in combination might explain why some older adults develop neurodegenerative diseases, while others retain their cognitive integrity until very old. What comes along with this, however, is the necessity for a cross‐validation of so far established results concerning the aging brain. Thus, the question that arises is whether independent samples of older adults that differ in demographics and lifestyle factors would still show similar association patterns between age, global and regional brain structure, and cognitive performance. While in the field of genetics, replication studies are already well established, it is not yet common practice in the field of neuroimaging. Therefore, the current study analyzed age‐related differences in brain structure and cognitive ability in two large independent but closely matched cohorts of older adults—both situated in central Europe—to explore how similar results are when using the same state‐of‐the‐art methodological protocols and what factors may explain potential between‐study differences.

Regarding brain structure, we used mean CT for the two hemispheres as a rough outcome measure. In addition to that, we decided to focus on brain regions that constitute the default mode network (DMN), a network that recently received much attention in aging research—especially with regard to functional connectivity (e.g., Hafkemeijer, van der Grond, & Rombouts, 2012). Because recent evidence from our group suggests a structural correlate for age differences in functional connectivity (Jockwitz et al., 2017), we were particularly interested to validate such first findings and assessed regional within‐network differences of the age‐brain structure relationships.

## 2 METHODS

Participants included in the current research project were recruited from two independent samples investigating brain–behavior relationships in older adults located in the larger Zurich area (Switzerland) and in the Ruhr district (Germany).

One sample comprised the ongoing Longitudinal Healthy Aging Brain (LHAB) database project at the University Research Priority Program “Dynamics of Healthy Aging” of the University of Zurich (Zollig et al., 2011). LHAB investigates age‐related dynamics of brain–behavior relationships in healthy older adults. A particular focus is placed on assessing and explaining interindividual variability in the observed aging trajectories, thus a broad spectrum of factors that supposingly influence such trajectories (i.e., lifestyle, sleep, and nutrition) is collected. In LHAB, older adults from Zurich and surrounding areas aged 65 and older (at baseline) are observed longitudinally with between‐measurement intervals of 1–2 years. Besides the eligibility requirements for the MR acquisition, further exclusion criteria were neurological and psychiatric diseases, a score on the Mini‐Mental State Examination of 26 and below and left handedness. LHAB participants are German native speakers or at least as proficient in German as it would be their native language. The study protocol was approved by the local Ethics Committee (Kantonale Ethikkommission Zurich). The initial sample of LHAB comprised 231 participants ranging from 64 to 87 years of age. Data acquisition in the LHAB project started in 2011. Currently, the data set covers an observation period of 4 years.

The second sample comprised 1000BRAINS at the Institute of Neuroscience and Medicine, Research Centre Jülich, a longitudinal population‐based study that assesses variability in brain structure and function during aging (Caspers et al., 2014). The 1000BRAINS sample is drawn from the 10‐year follow‐up cohort of the Heinz Nixdorf Recall Study, an epidemiological population‐based study of risk factors for atherosclerosis, cardiovascular disease, cardiac infarction, and death (Schmermund et al., 2002) and the affiliated MultiGeneration study. In 1000BRAINS, older adults aged 55 and older (at baseline) from the Heinz Nixdorf Recall study and their relatives (spouses and offspring; sampled from MultiGeneration study) are recruited, measured two times over a period of about 3–4 years. Exclusion from the study was dependent on the eligibility requirements for the MR acquisition based on the MR safety guidelines only (e.g., stents and heart pacemaker led to exclusion from the study). The study protocol was approved by the University of Duisburg‐Essen. The initial sample of 1000BRAINS comprised 1,317 participants ranging from 18 to 87 years of age.

For the aim of the current study, we focused on the first time point in both samples. Participants with missing values for the whole neuropsychological and/or brain data were excluded. Furthermore, participants were matched with respect to the age ranges in the two samples. Therefore, we first excluded 666 participants from 1000BRAINS being younger than 64 years of age. Afterward, we matched the two samples for gender and group size by randomly selecting the same number of participants within each age and gender group (64–69 years, 70–74 years, 75–79 years, and 80–85 years). This resulted in 228 participants for each of the two final samples: (a) LHAB: mean age: 70.7 years ± 4.9, 114 males, and 114 females; (b) 1000BRAINS: mean age: 70.7 ± 5.0 years, 114 males, and 114 females. For an overview of demographic variables of the two samples, see Table 1. Both studies assessed years of formal education as part of a structured anamnestic interview. In addition, all participants filled in a questionnaire concerning their physical and mental well‐being (LHAB: SF12; 1000BRAINS: SF36). In both samples, physical and mental health status scores (Ware, Keller, & Kosinski, 1995) were computed using only the SF12 items in order to assure comparability. Furthermore, global cognition was assessed in both samples. While participants from LHAB performed the Mini‐Mental State Examination (Folstein, Robins, & Helzer, 1983), participants from 1000BRAINS performed the DemTect in order to estimate a global cognitive status for each participant (Kalbe et al., 2004).

**Table 1:** Demographics of the two samples (1000BRAINS and LHAB). Mean values and *SD* of raw scores as well as group comparisons including *T* statistics, *p*‐values, and effect sizes

	1000BRAINS	LHAB	Levene test of equal variances (*F*/*p*‐value)	*T* test for equality of means (*T*/*p*‐value)	Cohen's *d*
Age (years)	70.69 ± 4.95	70.69 ± 4.89	0.056/0.814	−0.005/0.996	<0.001
Gender	114 m/114 f	114 m/114 f	NA	<0.001/1.00	<0.001
Education (years)	13.51 (±3.76)	14.66 (±3.43)	0.398/0.529	−3.40/0.001	0.320
Physical WB	48.69 (±8.10)	51.06 (±7.21)	4.44/0.036	−3.31/0.001	0.309
Mental WB	54.39 (±6.83)	55.06 (±5.84)	8.38/0.004	−1.12/0.263	0.105
Dementia screening	14.55 (±3.76) (DemTect)	28.83 (±1.02) (MMSE)	NA/NA	NA/NA	NA

LHAB = Longitudinal Healthy Aging Brain; WB = well‐being. *Note*. NA: not applicable since different tests were used that are not directly comparable.

### 2.1 Cognitive performance

Participants from both LHAB and 1000BRAINS took part in a large neuropsychological assessment consisting of tests in the domains attention, executive functions, working memory, episodic memory, and language functions. For comparison between the two samples, the following tasks were chosen: Trail Making Test (TMT; processing speed and concept shifting; Morris et al. (1989)), Leistungsprüfungssystem 50+ (LPS50+) Subtest 3 (reasoning; Sturm, Willmes, and Horn (1993)), Regensburger Wortflüssigkeitstest (RWT, semantic condition (verbal fluency); Aschenbrenner, Tucha, and Lange (2000)) and vocabulary tests (LHAB: Mehrfachwahl‐Wortschatz‐Intelligenztest (MWT‐B; Lehrl (2005)), 1000BRAINS: Wortschatztest (WST); Schmidt and Metzler (1992)). To extract comparable scores from the two vocabulary tests, we calculated the ratio between the total amount of words (MWT_B: 37 words; WST: 40 words) and the amount of correctly identified words. Since the selected neuropsychological tests were not normally distributed, all cognitive tests were first rank‐transformed and mean‐centered afterward before entering the statistical analysis. For a detailed test description, administration differences between samples and mean values per sample, see Table 2.

**Table 2:** Overview of administered cognitive test. Test description and differences in test administration (if applicable), raw scores, and *SD* of performance in the two samples as well as group comparisons including *T* statistics, *p*‐values, and effect sizes

	Test description	Differences in administration	1000BRAINS	LHAB	Levene test of equal variances (*F*/*p*‐value)	*T* test for equality of means (*T*/*p*‐value)	Cohen's *d*
**Processing speed (TMT A)**	Time (s) to connect randomly arranged digits in ascending order	–	43.48 (±15.71)	39.37 (±14.55)	1.85/0.175	−2.89/0.004	−0.271
**Concept shifting (TMT B–A)**	Time difference (s) between connecting alternately numbers and letters in ascending order (Part B) and randomly arranged digits in ascending order (Part A)	–	62.30 (±48.37)	53.79 (±28.13)	15.10/<0.001	−2.30/0.022	−0.215
**Reasoning (LPS50+, Subtest 3)**	Total number of correctly identified irregularities in rows of geometric figures within 5 min	–	19.37 (±5.14)	23.23 (±4.54)	3.30/0.070	−8.50/<0.001	0.796
**Verbal fluency (RWT, semantic)**	Total number of produced words belonging to a specific category in 2 min	1000BRAINS: category = jobs; overtly naming LHAB: category = animals; writing	22.26 (±6.71)	25.44 (±6.30)	1.49/0.224	−5.21/<0.001	0.489
**Vocabulary (WST/MWT‐B)**	Total number of correctly identified real words within rows of five pseudowords divided by total amount of rows	1000BRAINS: WST, LHAB: MWT‐B	0.76 (±0.14)	0.88 (±0.07)	51.18/<0.001	−12.10/<0.001	1.08

LHAB = Longitudinal Healthy Aging Brain; RWT = Regensburger Wortflüssigkeitstest; TMT = Trail Making Test; LPS50+ = Leistungsprüfungssystem 50+; WST = Wortschatztest; MWT = Mehrfachwahl‐Wortschatz‐Intelligenztest.

### 2.2 Data acquisition

For LHAB, data were acquired on a 3.0T Philips Ingenia scanner (Philips Medical Systems, Best, The Netherlands). T1‐weighted structural brain images were measured per visits with: TR = 8.18 ms, TE = 3.8 ms, flip angle = 8°, field of view (FoV) = 240 × 240 mm, isotropic voxel size = 1 × 1 × 1 mm, 160 slices per volume. For 1000BRAINS, data were acquired on a 3.0T Tim‐Trio MR scanner (Siemens Medical System, Erlangen, Germany). The T1‐weighted structural brain images were scanned per visit with: TR = 2.25 s, TE = 3.03 ms, flip angle = 9°, FoV = 256 × 256 mm, voxel resolution = 1 × 1 × 1 mm, 176 slices per volume. In both studies, T1‐imaging was part of a larger MR imaging protocol (see Caspers et al., 2014; Zollig et al., 2011).

### 2.3 Preprocessing

Anatomical images from both samples were preprocessed using the same automated surface‐based processing stream of the FreeSurfer Software package (version 6.0.0). For the LHAB sample, this was done via the FreeSurfer BIDS App (v6.0.0‐2; Gorgolewski et al. (2017). A detailed description of this pipeline is provided by Dale, Fischl, and Sereno (1999) as well as on http://surfer.nmr.mgh.harvard.edu. In short, the surface reconstruction pipeline includes (a) the segmentation of the structural brain images into gray matter, white matter, and cerebrospinal fluid, (b) motion correction, (c) intensity normalization, (d) transformation into Talairach space, (e) tessellation of gray/white matter boundary, and (f) correction of topological defects. The gray/white matter interface was then (g) expanded to create the pial surface (boundary between gray matter and cerebrospinal fluid), which finally consists of about 150,000 vertices per hemisphere with an average surface area of 0.5 mm^2^. Afterwards, (h) CT was calculated for each vertex as the shortest distance between the white matter surface and the corresponding vertex on the pial surface. No manual correction of the reconstructed surfaces (white matter, pial surface) was performed in the two studies.

For the purpose of the current study, mean measurements of CT per hemisphere were extracted from FreeSurfer (Fischl and Dale, 2000). In addition, CT was determined for six regions of interest belonging to the DMN, a bilateral network composed of the medial prefrontal cortex (anterior DMN), the posterior cingulate cortex/precuneus (medial posterior DMN) as well as the inferior parietal lobule (lateral posterior DMN). Those regions have been defined for the purpose of a previous study and are described in detail by Jockwitz et al. (2017). In short, functional resting state scans from 691 subjects in 1000BRAINS were preprocessed using the preprocessing pipeline provided by the FSL software package 5.0 (including denoising strategies: FIX; Griffanti et al. (2014); Salimi‐Khorshidi et al. (2014)). Afterwards, the DMN was extracted using an independent component analysis (ICA; MELODIC, implemented in FSL). To provide high reliability, this procedure was repeated 100 times (each sample consisted of 200 subjects). Finally, the resulting probability map was thresholded at 95% (using fslmaths, FSL) and binarized.

### 2.4 Statistical analysis

The purpose of the current study was to compare age‐related differences in cognitive abilities and CT in two large independent samples of older adults. Therefore, we first assessed general differences in sample characteristics (i.e., demographic variables), as well as cognitive abilities and CT (i.e., mean CT per hemisphere and regions of the DMN) using independent samples *T* tests. Thereafter, we assessed the following general linear models for each sample individually: (a) age‐related differences in CT, (b) age‐related differences in cognitive abilities, and (c) the relation between CT and cognitive abilities. To correct for possible factors that might influence the relation between age and cognitive abilities and CT, different models were set up including several covariates of no interest. The BASE model included the factors age and gender. The MAIN model was set up with age, gender, and education as factors, and the SENS (sensitivity) model included the factors age, gender, education as well as mental and physical well‐being. Results were corrected for multiple comparisons using the Bonferroni approach. To test whether trajectories of age‐related differences in the different dependent variables (cognitive abilities and CT) are comparable between the two samples, we calculated correlations between age and cognitive abilities and CT (while correcting for gender and education; MAIN) and compared them using Fisher's *Z* test (Eid, Gollwitzer, & Schmitt, 2011). Finally, in a supplementary analysis, we assessed age‐related differences in terms of cognitive performance and CT in a joint analysis (pooled samples), with additionally including “sample” as covariate (for results, see Supporting Information). The reason for this was an additional validation whether the results obtained by the “individual analyses” versus the “joint analysis” would be comparable in the current study.

## 3 RESULTS

When matching the two independent samples for age and gender, the two samples differed in both, demographic variables and cognitive performance. For raw scores and *T* statistics and Cohen's *d*, see Table 1 (Cohen's *d* < 0.5 = small; *d* < 0.8 = medium, and *d* > 0.8 = large). In more detail, participants from LHAB generally had a significantly higher formal education (years of education: *T* = −3.4; *p* = 0.001; *d* = 0.32) and higher physical well‐being (*T* = −3.31; *p* = 0.001; *d* = 0.31) as compared to participants from 1000BRAINS. Mental well‐being, however, did not differ between the two samples (*T* = −1.12; *p* = 0.263; *d* = 0.11).

With respect to cognitive abilities, we found that participants from LHAB showed better performance as compared to participants from 1000BRAINS in all psychometric tests assessed (processing speed: *T* = −2.89; *p* = 0.004; *d* = 0.271, concept shifting: *T* = −2.30; *p* = 0.022; *d* = 0.215, verbal fluency: *T* = −5.21; *p* < 0.001; *d* = 0.489, reasoning: *T* = −8.50; *p* < 0.001; *d* = 0.796 and vocabulary: *T* = −12.10; *p* < 0.001; *d* = 1.08; for detailed information, see Table 2).

When comparing structural brain metrics, we observed higher values for the participants from 1000BRAINS as compared to participants from LHAB, that is, total mean CT for right and left hemispheres, (right: *T* = 6.13; *p* < 0.001; *d* = 0.714; left: *T* = 7.62; *p* < 0.001; *d* = 0.574). The same was found for CT within the different parts of the DMN (left aDMN: *T* = 7.11; *p* < 0.001; *d* = 0.665; right aDMN: *T* = 5.02; *p* < 0.001; *d* = 0.470; left medial pDMN: *T* = 2.52; *p* = 0.012; *d* = 0.236; right medial pDMN: *T* = 4.79; *p* < 0.001; *d* = 0.448; left lateral pDMN: *T* = 6.93; *p* < 0.001; *d* = 0.649; right lateral pDMN: *T* = 4.48; *p* < 0.001, *d* = 0.420).

**Figure 1:**
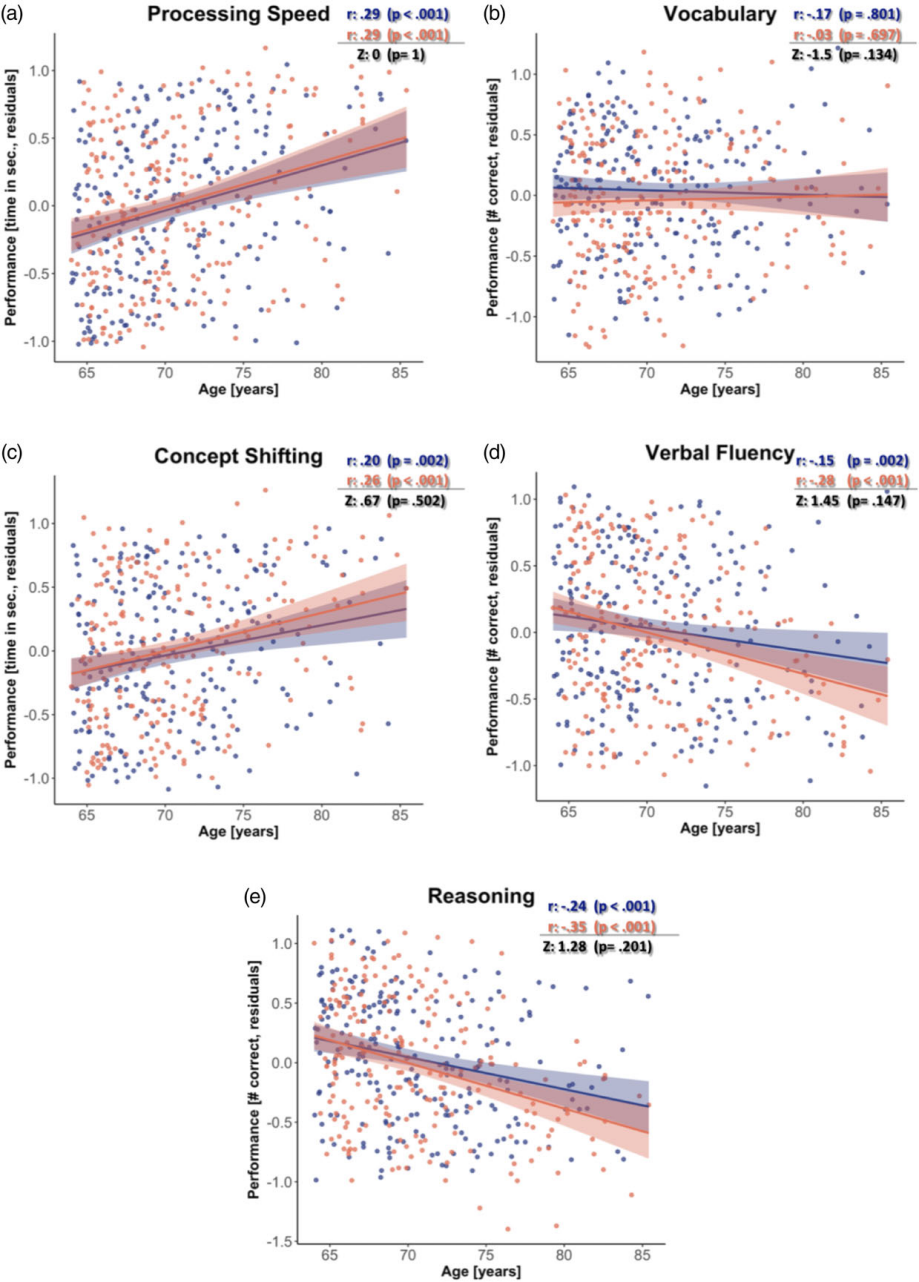
Relation between age and cognitive performance (residuals, corrected for gender and education) for the two samples, including regression lines, 95% confidence intervals, correlation coefficients, corresponding *p*‐values as well as the Fisher's *Z* test statistic and corresponding *p*‐value. 1000BRAINS is presented in blue and LHAB is presented in orange: (a) processing speed; (b) vocabulary; (c) concept shifting; (d) verbal fluency; and (e) problem solving. LHAB = Longitudinal Healthy Aging Brain [Color figure can be viewed at http://wileyonlinelibrary.com]

**Figure 2:**
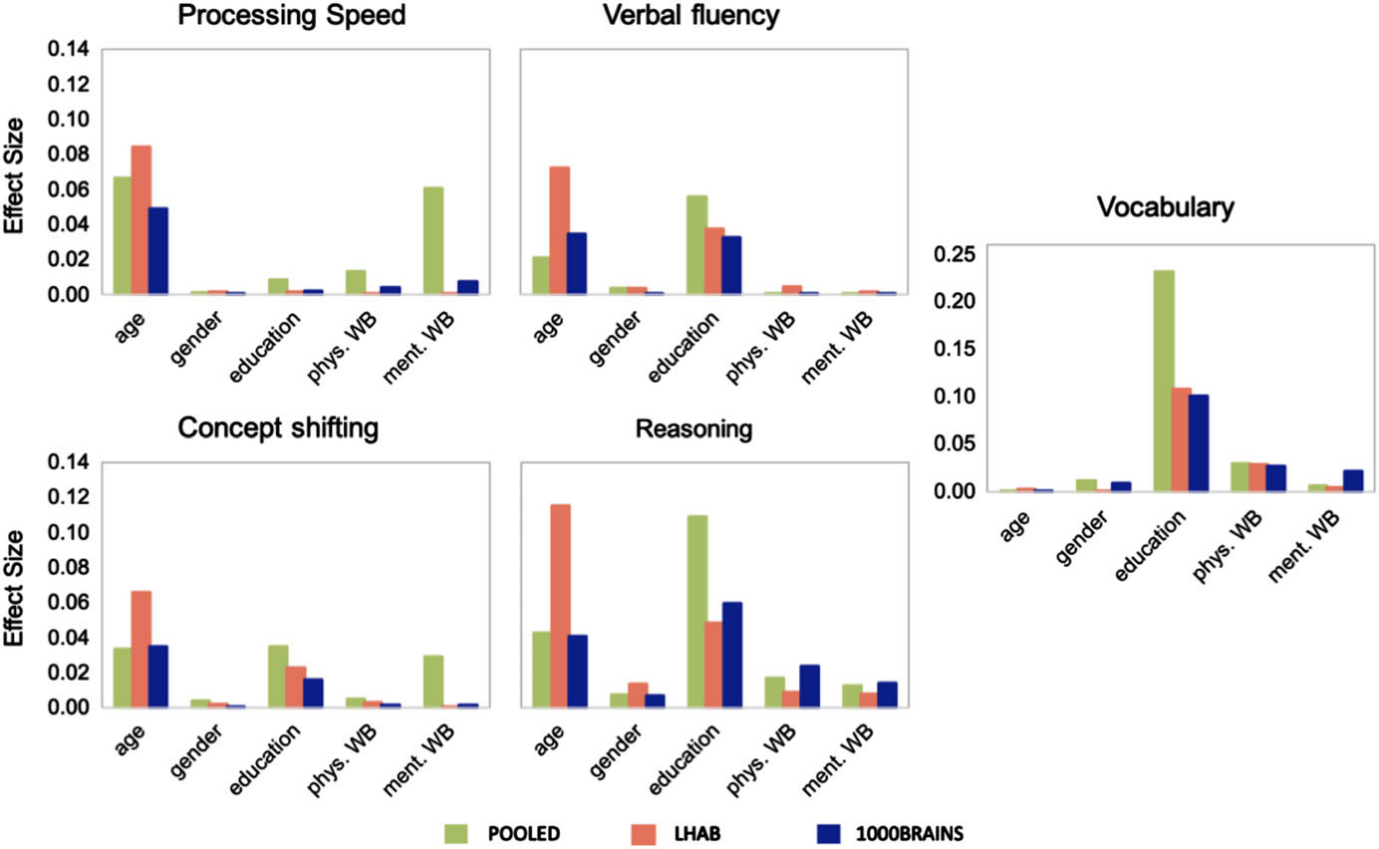
Profile plots of effect sizes (partial eta square) for cognitive performance with all covariates assessed: age, gender, education, physical WB, and mental WB 1000BRAINS are presented in blue, LHAB is presented in orange and the pooled data set is represented in green. LHAB = Longitudinal Healthy Aging Brain, WB = well‐being [Color figure can be viewed at http://wileyonlinelibrary.com]

In the following analyses, the relation between age and cognitive performance and CT, respectively, was assessed using different models (BASE, MAIN, and SENS). With respect to BASE (covariate: gender), we found age‐related differences for most of the cognitive tasks (i.e., lower cognitive performance in older adults). Effect sizes, measured using partial eta square were estimated as small to moderate (partial eta square is measured as the proportion of the total variance explained by the independent variable while correcting for the other independent variables, with partial eta square <0.01 is ranked as small; <0.06 as medium and >0.14 as large (Field, 2005; Richardson, 2011). Performance on the vocabulary tests remained stable across the ages in both samples. Almost all of these results remained significant in the MAIN model (covariates: age, gender, years of education; only exception: verbal fluency in 1000BRAINS did not survive correction for multiple comparisons) and in the SENS model (covariates: age, gender, years of education, mental well‐being, and physical well‐being; exceptions: verbal fluency and concept shifting did not survive correction for multiple comparisons). Importantly, age‐related differences were highly similar in the two samples (see Figure 1; results based on MAIN model: Fisher's *Z*: processing speed <0.001 [*p* = 0.251]; concept shifting = −0.67 [*p* = 0.503]; reasoning = 1.28 [*p* = 0.200]; verbal fluency = 1.45 [*p* = 0.147]; vocabulary = −1.5 [*p* = 0.134]). For profile plots showing the effects of the different covariates (age, gender, years of education, mental well‐being, and physical well‐being), see Figure 2. Table S1 (see Supporting Information) contains the detailed statistics for the age differences in cognitive performance and for the effects of the covariates of no interest (gender, years of education, mental, and physical well‐being).

**Figure 3:**
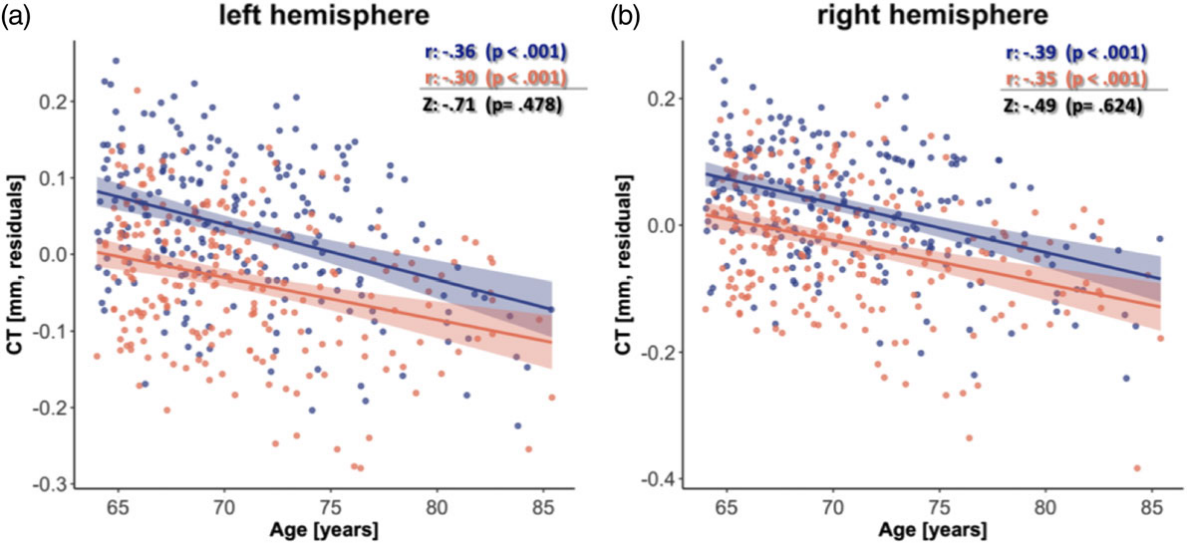
Relation between age and mean cortical thickness (residuals, corrected for gender and education) for the two samples, including regression lines, correlation coefficients, and corresponding *p*‐values, as well as the Steiger's *Z* test statistic and corresponding *p*‐value. 1000BRAINS is presented in blue and LHAB is presented in orange: (a) left hemisphere and (b) right hemisphere. LHAB = Longitudinal Healthy Aging Brain [Color figure can be viewed at http://wileyonlinelibrary.com]

**Figure 4:**
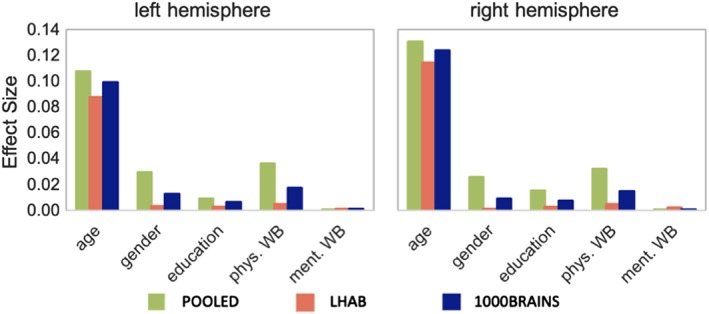
Profile plots of effect sizes (partial eta square) for mean cortical thickness with all covariates assessed: age, gender, education, physical WB, and mental WB 1000BRAINS are presented in blue, LHAB is presented in orange, and the pooled data set is represented in green. LHAB = Longitudinal Healthy Aging Brain, WB = well‐being [Color figure can be viewed at http://wileyonlinelibrary.com]

**Figure 5:**
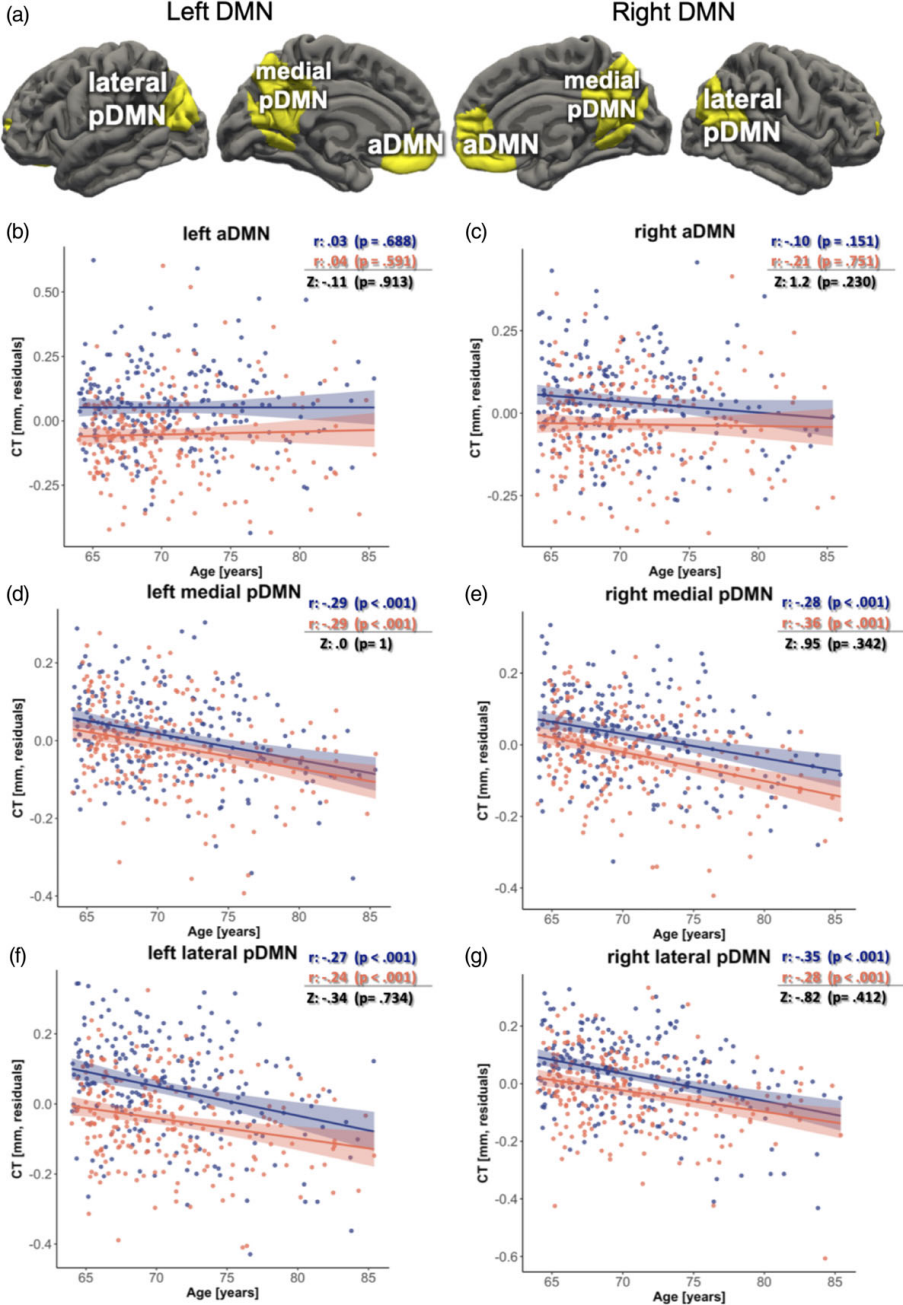
Relation between age and regional mean cortical thickness for parts of the DMN (residuals, corrected for gender and education) for the two samples, including regression lines, correlation coefficients and corresponding *p*‐values, as well as the Steiger's Z test statistic and corresponding *p*‐value. 1000BRAINS is presented in blue and LHAB is presented in orange: (a) DMN projected on a brain's surface consisting of the anterior (a)DMN (medial PFC), medial posterior (p)DMN (PCC and precuneus) and the lateral pDMN (caudal IPL); (b) left anterior (a)DMN; (c) right aDMN; (d) left medial posterior (p)DMN; (e) right medial pDMN; (f) left lateral pDMN; and (g) right lateral pDMN. LHAB = Longitudinal Healthy Aging Brain, PFC = prefrontal cortex, IPL = inferior parietal lobule, DMN = default mode network [Color figure can be viewed at http://wileyonlinelibrary.com]

**Figure 6:**
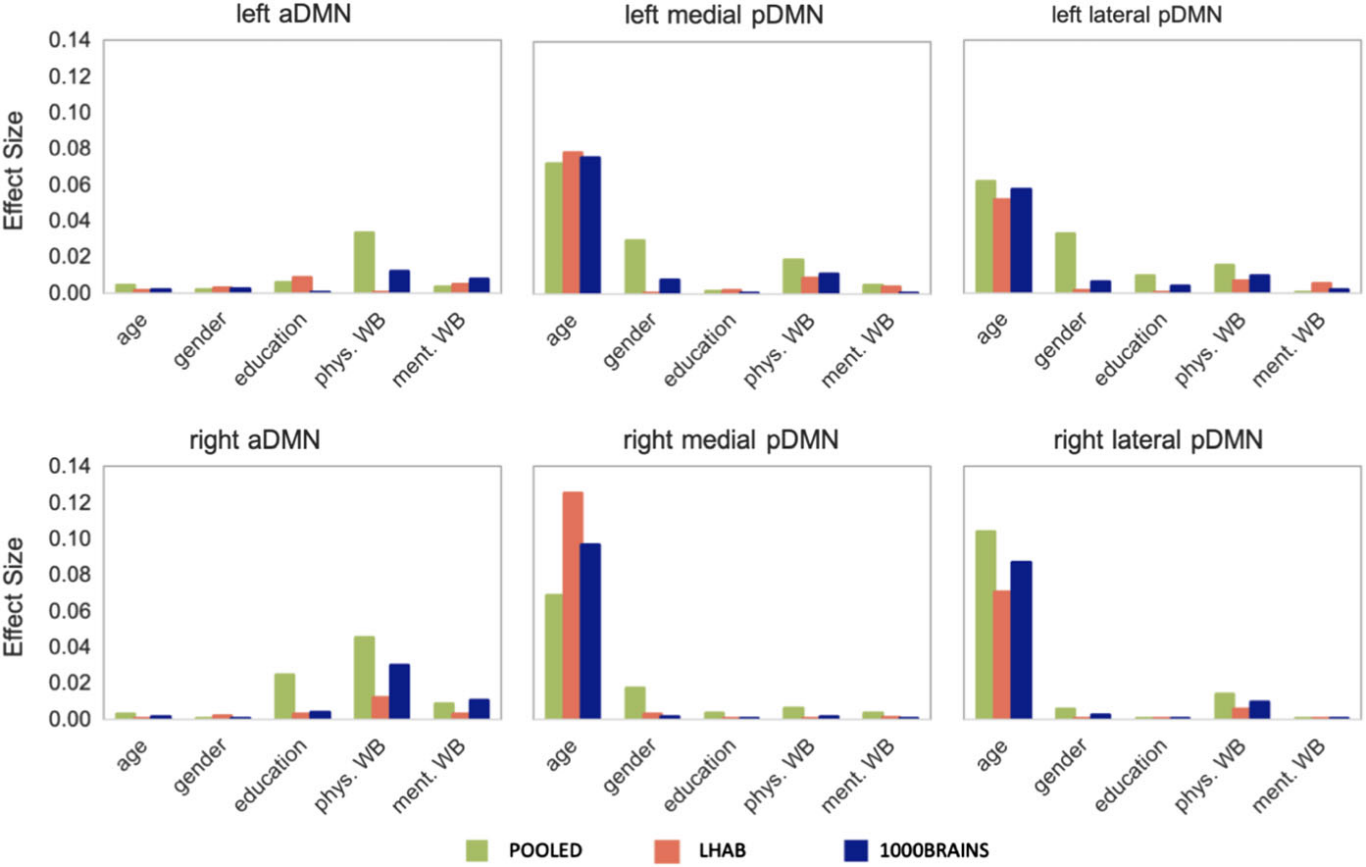
Profile plots of effect sizes (partial eta square) for regional mean cortical thickness (parts of the DMN) with all covariates assessed: age, gender, education, physical WB, and mental WB. 1000BRAINS are presented in blue, LHAB is presented in orange, and the pooled data set is represented in green. LHAB = Longitudinal Healthy Aging Brain, WB = well‐being, DMN = default mode network [Color figure can be viewed at http://wileyonlinelibrary.com]

In the second part of our analysis, we assessed age‐related differences in mean CT within left and right hemisphere (Figure 3, for effects sizes, see Figure 4, for statistics, see Table S2, Supporting Information), as well as parts of the DMN (see Figure 5; left and right: anterior DMN, medial posterior DMN, and lateral posterior DMN, for effect sizes, see Figure 6, for statistics, see Figure S3, Supporting Information). In our two samples, we find mean CT differences with age for the two hemispheres (left hemisphere: *F* = 33.24 [*p* < 0.001], right hemisphere: *F* = 40.15 [*p* < 0.001]; Table S2, see Supporting Information). With respect to regional differences in the association between CT and age, we found more pronounced age differences in CT for the posterior as compared to the anterior parts of the DMN (Table S3, see Supporting Information). For both samples, we found that for the left and right medial and lateral posterior DMN CT was smaller with higher age with a moderate effect size (partial eta square ranged from 0.07 to 0.12 in 1000BRAINS and from 0.08 to 0.13 in LHAB). Again, these effects were highly similar in the two samples (Fisher's *Z*: left medial posterior DMN = 0 [*p* = 1]; left lateral posterior DMN = −0.34 [*p* = 0.734]; right medial posterior DMN = −0.95 [*p* = 0.342]; right lateral posterior DMN = −0.82 [*p* = 0.412]; left anterior DMN = 0.11 [*p* = 0.913]; right anterior DMN = 1.2 [*p* = 0.230]).

Moreover, we assessed the relation between age (and other demographics), CT (of the DMN ROI's), and cognitive performance, with age and CT of the six ROIs being independent variables and cognitive performance being the dependent variable. Only the relations between age and cognitive performance (partial eta square ranged from 0.02 to 0.073 in 1000BRAINS and from 0.039 to 0.102 in LHAB) and education and cognitive performance (partial eta square ranged from 0.047 to 0.243 in 1000BRAINS and from 0.051 to 0.116 in LHAB) remained significant even when including all covariates into one model with small to moderate effect sizes. For all other factors, none of the analyses revealed significant results (after correction for multiple comparisons) in any of the two samples (Table S4; see Supporting Information).

In subsequent analyses, we also assessed age‐related differences of CT and cognitive abilities in a joint analysis for the two samples to additionally validate the results obtained by the individual analyses of the two samples. Here, again, the pooled sample showed age‐related differences in both, cognitive abilities (exception: vocabulary) as well as for the posterior parts of the DMN. In addition, the relation between CT and cognitive performance remained nonsignificant even when the two samples were analyzed in one statistical model. For a detailed overview of statistics, see Tables S1–S4 (Supporting Information).

Furthermore, assessing nonlinear effects of age (age^2^) on CT and cognitive performance (corrected for gender, education, physical, and mental well‐being) revealed no significant results after correction for multiple comparisons (for statistics, see Tables S5 and S6, Supporting Information). Finally, to rule out confounding of differences in data or surface reconstruction quality, we performed a supplementary analysis of the relation between age and CT in the DMN while including quality measurements (contrast to noise ratio for general data quality and Euler Numbers for quality of the surface reconstructions) as additional covariates to the SENS model. Age‐related differences in CT remained stable even when including these quality control parameters to the general linear model, that is, age‐related differences in CT for all posterior parts of the DMN but not the anterior DMN. For detailed statistics including group means and comparison, as well as general linear models, see Tables S7 and S8, Supporting Information.

Taken together, participants from LHAB seem to show a general superiority in cognitive performance as compared to participants from 1000BRAINS. However, the analysis of age‐related differences in cognitive performance and global and regional metrics of CT revealed similar results in both samples.

## 4 DISCUSSION

The present study assessed age‐related differences in cognitive abilities (processing speed, concept shifting, reasoning, verbal fluency, and vocabulary) and brain structure (measured by global and regional CT) in two closely matched samples of older adults. Despite significant differences in demographics between the two independent samples, we observed highly similar patterns of age‐related differences in both, cognitive abilities and brain structure, when using the same methodological approach.

### 4.1 Comparability of independent samples of older adults

In times of population aging, there is an increasing interest in assessing risk and protective factors that promote brain and cognitive health until old age. Especially in older adults, however, there is an enormous amount of variability between individuals regarding brain structure and cognitive abilities and the “biological age” does not prove itself sufficient to explain this variability (Goh & Park, 2009; Park & Reuter‐Lorenz, 2009; Reuter‐Lorenz & Cappell, 2008; Reuter‐Lorenz & Lustig, 2005; Reuter‐Lorenz & Park, 2014). Previous research rather suggests that interindividual differences in variables such as education, lifestyle habits, or genetic markers should be taken into consideration to explain why some older adults exhibit decline (up to developing neurodegenerative diseases), while others are able to retain their level of functioning until old age (Barnard et al., 2014; Kohncke et al., 2016; Laukka et al., 2013; Lovden et al., 2017; Raz et al., 2005; van Hooren et al., 2007). The problem with identifying such factors is that single risk or protective factors only explain small parts of the interindividual variance regarding cognitive performance and brain structure in the older adult population, which necessitates large sample sizes to increase statistical power to uncover these small effects (Button et al., 2013). One promising approach here is the pooling of existing data, that is, the joint analysis of different samples. Data pooling with different samples covering the whole adult age range revealed age‐related differences in terms of CT (Dickerson et al., 2008; Fjell et al., 2009; Jahanshad & Thompson, 2017; Jovicich et al., 2013). However, one has to keep in mind that data pooling across different study populations, might lead to an intermixture of sample‐specific biological as well methodological variability which might result in an absence of effects, especially when assessing heterogeneous populations such as older adults. Differences in demographics, methods applied as well as scanner variability have been proposed to be main factors that lead to the heterogeneity of results in terms of brain structure and function in older adults in the field of neuroscience (Afonso et al., 2017; Han et al., 2006; Hanggi et al., 2015; Jancke, Merillat, Liem, & Hanggi, 2015; Kohncke et al., 2016; Liem et al., 2015; Lovden et al., 2017; Trachtenberg et al., 2012). The two samples used in the current study, LHAB and 1000BRAINS, represent such heterogeneous study populations consisting of older adults. Therefore, in the interest of the current study, we individually characterized and compared two different independent samples in terms of demographics, cognitive abilities, and their relationship with brain aging.

LHAB has its focus on healthy older adults excluding participants with a history of neurological diseases and cognitive impairment (Zollig et al. (2011). On the other hand, 1000BRAINS is conducted as a population‐based epidemiological cohort study, excluding subjects only if they do not meet the eligibility requirements for the MR acquisition based on the MR safety guidelines (Caspers et al. (2014). Thus, although in the current study, we assured that the two samples would not differ in their age ranges and gender distribution, the two samples differed in several sample characteristics. Participants from LHAB on average had more years of education, as well as a higher physical well‐being as compared to participants from 1000BRAINS. This result is completely in line with the observations made by the Organization for Economic Co‐operation and Development, namely that Switzerland compared to Germany is constantly ranked as being superior in terms of job income and quality, health, life satisfaction, as well as environmental and community factors (http://www.oecdbetterlifeindex.org/countries/switzerland/)).

In line with the predictions of the scaffolding theory of cognitive aging (Goh & Park, 2009; Park & Reuter‐Lorenz, 2009; Reuter‐Lorenz & Park, 2014), higher education as well as engagement in physical activities (which seems to be related to higher physical well‐being as tested in the current studies; Bize, Johnson, and Plotnikoff (2007)) have repeatedly been shown to protectively influence the neurocognitive aging process. Both have been related to higher cognitive functioning and less brain atrophy during normal as well as pathological aging, such as mild cognitive impairment and Alzheimer's disease (Afonso et al., 2017; Amieva et al., 2014; Miller, Taler, Davidson, & Messier, 2012; Ritchie, Bates, Der, Starr, & Deary, 2013; Schneeweis, Skirbekk, & Winter‐Ebmer, 2014; Sofi et al., 2011; Tucker‐Drob, Johnson, & Jones, 2009; Zahodne et al., 2011). It is therefore plausible that participants from LHAB showed superior performances in all cognitive tests assessed (processing speed, concept shifting, reasoning, verbal fluency, and vocabulary).

The comparison of CT between the two samples, however, revealed higher global as well as regional CT values for participants from 1000BRAINS. This result seems counterintuitive at first sight. Based on the sample differences in terms of demographics and cognitive abilities, one would have predicted participants from LHAB to show thicker cortices given that the pertinent literature tends to show positive associations between cognitive ability and the amount of gray matter as measured with CT and gray matter volume or density in the aging population (for an overview, see, e.g., Harada et al., 2013). From our view, the most likely explanation is that these sample differences in CT are due to the different MR scanners used. It has been shown before that even when assessing structural 3D brain images from one and the same person, CT values, but also other metrics, such as brain volume differ between the different scanners (Bauer, Jara, Killiany, & Alzheimer's Disease Neuroimaging, 2010; Dickerson et al., 2008; Fortin et al., 2017; Han et al., 2006; Kruggel, Turner, Muftuler, & Alzheimer's Disease Neuroimaging, 2010; Schlett et al., 2016; Stonnington et al., 2008; Westlye et al., 2009). Thus, direct comparisons of brain metrics between different samples should only be executed with caution.

### 4.2 Generalizability of age‐related differences in cognitive abilities and CT

Within the scope of the current study, we decided to separately analyze the associations between age and brain structure and cognitive abilities in the two samples and compared the resulting associations using Fisher's *Z*. Although the two samples differed regarding both, cognitive performance and CT, we revealed highly similar slopes for age‐related differences in global as well as regional CT. In line with preceding studies examining global CT, higher age was associated with lower mean CT in both hemispheres for the two samples (Lemaitre et al., 2012; Long et al., 2012; Salat et al., 2004). Similarly, the age‐effect patterns found for cognitive ability did not differ across samples. Higher age was associated with lower cognitive functioning in all cognitive tasks assessed, except in the vocabulary test, where no significant relationship was revealed between age and ability scores.

The similarity of the cross‐sectional age‐effect patterns that we observe across LHAB and 1000BRAINS indicates that the lower level of education or physical well‐being evident in 1000BRAINS does not considerably enhance age differences (i.e., steeper slope in 1000BRAINS sample). Put into the context of cognitive reserve, the between‐sample differences in cognitive ability together with the similarity of age slopes, may suggest that participants from LHAB (with a higher education and higher physical well‐being) reach the criterion for cognitive impairment later as compared to participants from 1000BRAINS, primarily because they started off at higher levels of cognitive ability. However, by means of the presently used cross‐sectional data sets, this proposition cannot be tested on the level of individual trajectories. More empirical studies in the field of cognitive reserve and longitudinal changes of cognitive abilities are necessary to shed more light on the role of cognitive reserve—and education as one important proxy of it—in defining the rate of cognitive decline (for a recent review, see Christie et al. (2017)).

To explore in more detail whether the relationship between age and cognitive performance/CT would be differentially influenced by the different covariates (education, physical, and mental well‐being) in the two samples, we set up different statistical models (BASE, MAIN, and SENS). Although the different covariates seemed to explain different amounts of variance in the cognitive abilities/CT in the two samples, age‐related differences in cognition/CT remained highly similar across samples. For example, mental well‐being had a significant influence on processing speed for the sample of 1000BRAINS, but not LHAB. Nevertheless, this difference obviously did not have a considerable impact on the age‐related differences in processing speed. Thus, while education and physical well‐being might influence the general level of cognitive performance, it seems that these age‐related differences seem to be robust against the possible influences tested in the current samples.

### 4.3 Regional differences in CT

Beyond assessing mean CT for the two hemispheres, we also analyzed age‐related differences in regional CT (different parts of the DMN). The choice of regions of interest was based on an earlier study of Jockwitz et al. (2017). Herein, the authors aimed at assessing structural correlates for functionally established theories of the aging brain. In detail, it has been shown that during performance of a memory task (but also in the resting state), older in comparison to younger adults, show stronger activation/connectivity patterns in the more anterior parts of the DMN. At the same time, activation patterns in the more posterior parts of the DMN were reported to be stronger in younger compared to older participants. Thus, with increasing age, there seems to be a shift in brain activation patterns from more posterior to more anterior brain regions (posterior to anterior shift in aging [PASA]) that helps to maintain cognitive performance as stable as possible (Davis, Dennis, Daselaar, Fleck, & Cabeza, 2008; Jones et al., 2011).

In the current study, we exemplarily used the parts of the DMN to assess regional generalizability of age‐related differences in brain structure and found age‐related decreases in CT for all the posterior parts of the DMN in both samples. In contrast to that, the anterior parts of the DMN did not show age‐related differences in any of the two samples. This finding supports a previous study by Jockwitz et al. (2017), in which the authors presented a structural correlate for the posterior to anterior shift in activation patterns, namely a more pronounced decrease in cortical folding for the posterior parts of the DMN as compared to the more anterior parts of the DMN in a sample of older adults (1000BRAINS). Moreover, the current results extend previous results by showing that structural correlates for PASA can also be generalized over different independent samples of older adults with different demographical characteristics and different brain metrics used (local gyrification index vs. CT).

### 4.4 Brain–behavior associations

In the current study, the associations between cognitive abilities and CT were weak and did not survive correction for multiple comparisons. This result was stable over the different statistical models used (BASE, MAIN, SENS) as well as for the different samples (1000BRAINS, LHAB, pooled sample of the two). This result is in line with previous studies showing only weak associations between brain structure and cognitive performance, especially when examining older adults (e.g., Gunning‐Dixon & Raz, 2000; de Mooij, Henson, Waldorp, & Kievit, 2017). This, in turn accords with the scaffolding theory of aging stating that intraindividual regulatory processes (e.g., changes in functional connectivity) within older adults might compensate for structural brain decline thereby keeping cognitive abilities relatively stable (Reuter‐Lorenz & Park, 2014). Thus, in the current study, the relation between CT and cognitive abilities were expected to be rather weak. To explore this in more detail, further longitudinal studies are warranted that assess both, structural as well as functional changes in the course of aging in relation to intraindividual changes in cognitive abilities.

Another reason, especially when comparing the current results to the results reported in Jockwitz et al. (2017) for an absence of significant relationships between cognitive abilities and CT could be due differences in structural brain metrics used. The aforementioned study of Jockwitz et al. (2017) used the local gyrification index as measure for cortical atrophy in the regions of interest, measuring the complexity of the brain composed of gray matter and structural connectivity. The current study used CT as measure for cortical atrophy, since this is one of the most often used brain metrics to study the effects of age on brain structure. CT measures rather local gray matter differences only. Thus, different structural brain metrics might result in different results. Beyond that, it might still be possible that the chosen regions of interest (DMN) might not be directly related to performance in the neuropsychological tests assessed. Beyond that, it might still be possible that the chosen regions of interest (DMN) might not be directly related to performance in the neuropsychological tests assessed. Although parts of the DMN have previously been associated with attention and executive functions, other tests, which were not available for the two samples, might be interesting to investigate in this context for example, episodic memory function. And finally, larger sample sizes might be necessary to obtain small but significant results, as it has been the case in the aforementioned study of Jockwitz et al. (2017); *n* = 749.

### 4.5 Pooled versus individual analyses

In the current study, we decided not to pool data of the two samples but to analyze the samples individually with respect to age‐related differences in cognitive performance and brain structure. While the results were highly similar for the cross‐sectional age trajectories in terms of CT and cognitive performance, differences were found for the relation between the other covariates included in the models (i.e., SENS) and cognitive performance and CT, respectively. For example, when looking at 1000BRAINS, we found a moderate effect of mental well‐being on processing speed. On the other hand, for LHAB and for the pooled sample, there was no effect of mental well‐being on processing speed. These distinct outcomes might be the result of differences in sample characteristics. The sample of 1000BRAINS is a population‐based sample. In contrast to that, the LHAB study only included participants without any neurological and psychiatric diseases and a score on the Mini‐Mental State Examination of at least 26. These sample characteristics might be one explanation why mental well‐being plays a significant role in terms of cognitive performance differences in 1000BRAINS but not in LHAB. Previous studies often assessed age as independent factor in pooled data analyses consisting of older adults (e.g., Fjell et al. (2009)). In the current study, we could show that age revealed the strongest effects on both cognitive performance and CT, and this seems to be highly similar even in independent samples of older adults. Thus, for such robust effects data pooling might be a good option to increase sample sizes and statistical power (Button et al., 2013). However, other risk and protective factors on the aging brain (such as mental well‐being) might be study specific, depending on the sample characteristics. Following, when samples are highly heterogeneous, a pooled analysis might underestimate such influences. A combination of both, pooled and individual analyses seem to be an optimal solution to explore influencing factors on the aging brain.

### 4.6 Limitations and future directions

The study has several advantages as well as limitations which should be addressed. First, the current study investigated CT as one metric of brain structure. CT is a popular and sensitive metric in the frame of age‐related differences or changes in gray matter, for example, see Fjell et al. (2015, 2009, 2013); Hogstrom et al., 2013. Given the upcoming trend in data pooling procedures, we thought that CT would therefore be of interest in the current cross‐validation study. Nevertheless, in future research, other estimated of gray and white matter as well as functional connectivity should be validated between independent studies, to further evaluate the generalizability of results and advantages and disadvantages of data pooling procedures. Second, with respect to the current study, we decided to match the two samples with respect to age and gender distributions and compare the correlations using Fisher's *Z*. For the future, we suggest to further evaluate different methodological approaches when cross‐validating independent samples with regard to brain metrics and or cognitive functions. First, different matching procedures should be investigated and compared. For example, future studies could not only match samples with regard to age and gender, but also with respect to cognitive functioning using propensity score matching. Furthermore, it would be useful to evaluate other statistical methods to cross validate age‐related differences in brain structure and cognitive performance, especially when examining more than two samples. Finally, future studies should explore the importance of covariates. Since the choice of covariates to include into statistical models is highly variable across studies (see Silberzahn et al., 2017), future research should investigate this topic more intensively. For example, the current study assessed education as one indicator for socioeconomic status. Since socioeconomic status includes more than education, for example, occupation and income, future research should also assess other indicators and investigate the influence of these factors on cognition and brain structure.

Moreover, we are aware of the fact that scanner differences might contribute to the differences in sample means in terms of CT in the current study. One way to systematically explore this would be a traveling phantom that can be used to assess scanner differences. The current analyses investigated two independent samples of already completed measurements. Therefore, a retrospective methodical validation was not feasible. However, we would suggest such quality control measurement for future studies with planned study comparisons.

Finally, we have to mention that PASA is just one explanation for the results found in the current study. However, differences in image quality between anterior and posterior parts of the brain might be also responsible for the findings on age‐related differences in CT. Future studies should be designed to systematically investigate between‐subject variability across the different regions of the brain, its sources (i.e., measurement quality) and implications for analysis of data resulting from regions with differing variability.

## 5 CONCLUSIONS

Taken together, the current results show that when comparing age‐related differences in cognitive abilities and CT in two different and independent samples within the same age range and composed of the same gender distribution, age‐related differences in cognitive performance as well as global and regional CT can be generalized over different samples, assuming the same methodology is used. While data pooling has the advantage to increase statistical power to uncover small effects in the aging population, the current results show the usefulness of conducting separate analyses across samples consisting of distinct study populations, with comparison of the overall trends obtained in each analysis. Future multicenter studies and imaging consortia might at least use a combination of the two approaches to unravel the complexity of the aging brain in its entirety.

## Supporting information


**Table S1** Overview of general linear models assessing age‐related differences in cognitive abilities for each sample individually (1000BRAINS/LHAB/pooled analysis): Results presented for the three different models: BASE, MAIN, and SENS, with *F*‐values (*p*‐values/partial eta square). Significant results (*p* < 0.05) are written in bold; significant results even after correcting for multiple comparisons for the two samples 1000BRAINS and LHAB (five cognitive tests × 2 samples × three models [*p* = 0.05/30]) are marked in yellow.
**Table S2**: Overview of general linear models assessing age‐related differences in mean cortical thickness of the left and right hemisphere for each sample individually (1000BRAINS/LHAB/pooled analysis): Results presented for the three different models: BASE, MAIN, and SENS, with *F*‐values (*p*‐values/partial eta square). Significant results (*p* < 0.05) are written in bold; significant results even after correcting for multiple comparisons for the two samples 1000BRAINS and LHAB (two hemispheres × two samples × three models [*p* = 0.05/12]) are marked in yellow.
**Table S3**: Overview of general linear models assessing age‐related differences in mean cortical thickness of the different parts of the default mode network for each sample individually (1000BRAINS/LHAB/pooled analysis): Results presented for the three different models: BASE, MAIN, and SENS, with *F*‐values (*p*‐values/partial eta square). Significant results (*p* < 0.05) are written in bold; significant results even after correcting for multiple comparisons for the two samples 1000BRAINS and LHAB (six regions of interest × two samples × three models [*p* = 0.05/36]) are marked in yellow.
**Table S4**: Overview of general linear models assessing the relation between cognitive performance and mean cortical thickness of the different parts of the default mode network for each sample individually (1000BRAINS/LHAB/pooled analysis): Results presented for the three different models: BASE, MAIN, and SENS, with *F*‐values (*p*‐values/partial eta square). Significant results (*p* < 0.05) are written in bold; significant results even after correcting for multiple comparisons for the two samples 1000BRAINS and LHAB (six regions of interest × two samples × three models [*p* = 0.05/36]) are marked in yellow.
**Table S5**: Relation between age^2^ and cognitive performance (SENS + age^2^) with *T*‐values and *p*‐values in brackets.
**Table S6**: Relation between age^2^ and cortical thickness for the different ROIs of the default mode network (SENS + age^2^) with *T*‐values and *p*‐values in brackets.
**Table S7**: Quality control (QC) measurements of structural brain images (CNR) and surface reconstruction (Euler No.), both calculated using FreeSurfer. Independent sample *t* tests revealed significant differences in both, data quality as well as surface reconstruction quality, with the LHAB sample being superior in terms of both.
**Table S8**: Relation between age and cortical thickness for the different parts of the default mode network (DMN), when including QC measurements (left and right hemispheric: Euler NO, CNR for gray/white interface, CNR for gray matter/CSF) as covariate into the statistical model (SENS + QC measurements). Age‐related differences in CT remain stable, that is, no significant age‐related differences for the anterior parts of the DMN, while all posterior parts show significant age‐related decreases in CT.

## References

[hbm24524-bib-0001] Afonso, R. F. , Balardin, J. B. , Lazar, S. , Sato, J. R. , Igarashi, N. , Santaella, D. F. , … Kozasa, E. H. (2017). Greater cortical thickness in elderly female yoga practitioners—A cross‐sectional study. Frontiers in Aging Neuroscience, 9, 201 10.3389/fnagi.2017.00201 28676757 PMC5476728

[hbm24524-bib-0002] Amieva, H. , Mokri, H. , Le Goff, M. , Meillon, C. , Jacqmin‐Gadda, H. , Foubert‐Samier, A. , … Dartigues, J. F. (2014). Compensatory mechanisms in higher‐educated subjects with Alzheimer's disease: A study of 20 years of cognitive decline. Brain, 137(Pt. 4), 1167–1175. 10.1093/brain/awu035 24578544

[hbm24524-bib-0003] Aschenbrenner, A. , Tucha, O. , & Lange, K. (2000). RWT Regensburger Wortflüssigkeits‐Test. Göttingen, Germany: Hogrefe.

[hbm24524-bib-0004] Bamberg, F. , Kauczor, H.‐U. , Weckbach, S. , Schlett, C. L. , Forsting, M. , Ladd, S. C. , … Investigators . (2015). Whole‐body MR imaging in the German National Cohort: Rationale, design, and technical background. Radiology, 277(1), 206–220. 10.1148/radiol.2015142272 25989618

[hbm24524-bib-0005] Barnard, N. D. , Bush, A. I. , Ceccarelli, A. , Cooper, J. , de Jager, C. A. , Erickson, K. I. , … Squitti, R. (2014). Dietary and lifestyle guidelines for the prevention of Alzheimer's disease. Neurobiology of Aging, 35(Suppl. 2), S74–S78. 10.1016/j.neurobiolaging.2014.03.033 24913896

[hbm24524-bib-0006] Bauer, C. M. , Jara, H. , Killiany, R. , & Alzheimer's Disease Neuroimaging, I . (2010). Whole brain quantitative T2 MRI across multiple scanners with dual echo FSE: Applications to AD, MCI, and normal aging. NeuroImage, 52(2), 508–514. 10.1016/j.neuroimage.2010.04.255 20441797 PMC2907072

[hbm24524-bib-0007] Bize, R. , Johnson, J. A. , & Plotnikoff, R. C. (2007). Physical activity level and health‐related quality of life in the general adult population: A systematic review. Preventive Medicine, 45(6), 401–415. 10.1016/j.ypmed.2007.07.017 17707498

[hbm24524-bib-0008] Button, K. S. , Ioannidis, J. P. , Mokrysz, C. , Nosek, B. A. , Flint, J. , Robinson, E. S. , & Munafo, M. R. (2013). Power failure: Why small sample size undermines the reliability of neuroscience. Nature Reviews Neuroscience, 14(5), 365–376. 23571845 10.1038/nrn3475

[hbm24524-bib-0009] Caspers, S. , Moebus, S. , Lux, S. , Pundt, N. , Schutz, H. , Muhleisen, T. W. , … Amunts, K. (2014). Studying variability in human brain aging in a population‐based German cohort‐rationale and design of 1000BRAINS. Frontiers in Aging Neuroscience, 6, 149 10.3389/fnagi.2014.00149 25071558 PMC4094912

[hbm24524-bib-0010] Christie, G. J. , Hamilton, T. , Manor, B. D. , Farb, N. A. S. , Farzan, F. , Sixsmith, A. , … Moreno, S. (2017). Do lifestyle activities protect against cognitive decline in aging? A review. Frontiers in Aging Neuroscience, 9, 381 10.3389/fnagi.2017.00381 29209201 PMC5701915

[hbm24524-bib-1011] Dale, A. M. , Fischl, B. , & Sereno, M. I. (1999). Cortical surface‐based analysis. I. Segmentation and surface reconstruction. NeuroImage, 9(2), 179–194. 9931268 10.1006/nimg.1998.0395

[hbm24524-bib-0011] Davis, S. W. , Dennis, N. A. , Daselaar, S. M. , Fleck, M. S. , & Cabeza, R. (2008). Que PASA? The posterior‐anterior shift in aging. Cerebral Cortex, 18(5), 1201–1209. 17925295 10.1093/cercor/bhm155 PMC2760260

[hbm24524-bib-1010] de Mooij, S. M. , Henson, R. N. , Waldorp, L. J. , & Kievit, R. A. (2018). Age differentiation within grey matter, white matter and between memory and white matter in an adult lifespan cohort. Journal of Neuroscience, 1627–1617. 10.1523/JNEUROSCI.1627-17.2018 PMC6010564 29848485

[hbm24524-bib-0012] Dickerson, B. C. , Fenstermacher, E. , Salat, D. H. , Wolk, D. A. , Maguire, R. P. , Desikan, R. , … Fischl, B. (2008). Detection of cortical thickness correlates of cognitive performance: Reliability across MRI scan sessions, scanners, and field strengths. NeuroImage, 39(1), 10–18. 10.1016/j.neuroimage.2007.08.042 17942325 PMC2141650

[hbm24524-bib-0013] Dickie, D. A. , Job, D. E. , Gonzalez, D. R. , Shenkin, S. D. , Ahearn, T. S. , Murray, A. D. , & Wardlaw, J. M. (2013). Variance in brain volume with advancing age: Implications for defining the limits of normality. PLoS One, 8(12), e84093. 24367629 10.1371/journal.pone.0084093 PMC3868601

[hbm24524-bib-0015] Eid, M. , Gollwitzer, M. , & Schmitt, M. (2011). Statistik und Forschungsmethoden Lehrbuch. Weinheim,. Germany: Beltz.

[hbm24524-bib-0016] Field, A. (2005). Exploring data in discovering statistics using SPSS (Vol. 2, pp. 63–106). London, England: Sage Publications.

[hbm24524-bib-1009] Fischl, B. , Sereno, M. I. , & Dale, A. M. (1999). Cortical surface‐based analysis. II: inflation, flattening, and a surface‐based coordinate system. NeuroImage, 9(2), 195–207. 9931269 10.1006/nimg.1998.0396

[hbm24524-bib-1008] Fjell, A. M. , Grydeland, H. , Krogsrud, S. K. , Amlien, I. , Rohani, D. A. , Ferschmann, L. , … Walhovd, K. B. (2015). Development and aging of cortical thickness correspond to genetic organization patterns. Proceedings of the National Academy of Sciences of the United States of America, 112, 15462–15467. 26575625 10.1073/pnas.1508831112 PMC4687601

[hbm24524-bib-0017] Fjell, A. M. , McEvoy, L. , Holland, D. , Dale, A. M. , Walhovd, K. B. , & Alzheimer's Disease Neuroimaging, I . (2014). What is normal in normal aging? Effects of aging, amyloid and Alzheimer's disease on the cerebral cortex and the hippocampus. Progress in Neurobiology, 117, 20–40. 10.1016/j.pneurobio.2014.02.004 24548606 PMC4343307

[hbm24524-bib-0018] Fjell, A. M. , Walhovd, K. B. , Reinvang, I. , Lundervold, A. , Salat, D. , Quinn, B. T. , … Dale, A. M. (2006). Selective increase of cortical thickness in high‐performing elderly‐‐structural indices of optimal cognitive aging. NeuroImage, 29(3), 984–994. 10.1016/j.neuroimage.2005.08.007 16176876

[hbm24524-bib-0019] Fjell, A. M. , Westlye, L. T. , Amlien, I. , Espeseth, T. , Reinvang, I. , Raz, N. , … Walhovd, K. B. (2009). High consistency of regional cortical thinning in aging across multiple samples. Cerebral Cortex, 19(9), 2001–2012. 10.1093/cercor/bhn232 19150922 PMC2733683

[hbm24524-bib-0020] Fjell, A. M. , Westlye, L. T. , Grydeland, H. , Amlien, I. , Espeseth, T. , Reinvang, I. , … Alzheimer Disease Neuroimaging, I . (2013). Critical ages in the life course of the adult brain: Nonlinear subcortical aging. Neurobiology of Aging, 34(10), 2239–2247. 10.1016/j.neurobiolaging.2013.04.006 23643484 PMC3706494

[hbm24524-bib-0021] Fjell, A. M. , Westlye, L. T. , Grydeland, H. , Amlien, I. , Espeseth, T. , Reinvang, I. , … Alzheimer Disease Neuroimaging, I . (2014). Accelerating cortical thinning: Unique to dementia or universal in aging? Cerebral Cortex, 24(4), 919–934. 10.1093/cercor/bhs379 23236213 PMC3948495

[hbm24524-bib-1007] Folstein, M. F. , Robins, L. N. , & Helzer, J. E. (1983). The mini‐mental state examination. Archives of general psychiatry, 40(7), 812–812. 6860082 10.1001/archpsyc.1983.01790060110016

[hbm24524-bib-0022] Fortin, J. P. , Cullen, N. , Sheline, Y. I. , Taylor, W. D. , Aselcioglu, I. , Cook, P. A. , … Shinohara, R. T. (2017). Harmonization of cortical thickness measurements across scanners and sites. NeuroImage, 167, 104–120. 10.1016/j.neuroimage.2017.11.024 29155184 PMC5845848

[hbm24524-bib-0023] German National Cohort, C. (2014). The German National Cohort: Aims, study design and organization. European Journal of Epidemiology, 29(5), 371–382. 10.1007/s10654-014-9890-7 24840228 PMC4050302

[hbm24524-bib-0024] Goh, J. O. , & Park, D. C. (2009). Neuroplasticity and cognitive aging: The scaffolding theory of aging and cognition. Restorative Neurology and Neuroscience, 27(5), 391–403. 19847066 10.3233/RNN-2009-0493 PMC3355626

[hbm24524-bib-0025] Gorgolewski, K. J. , Alfaro‐Almagro, F. , Auer, T. , Bellec, P. , Capota, M. , Chakravarty, M. M. , … Poldrack, R. A. (2017). BIDS apps: Improving ease of use, accessibility, and reproducibility of neuroimaging data analysis methods. PLoS Computational Biology, 13(3), e1005209 10.1371/journal.pcbi.1005209 28278228 PMC5363996

[hbm24524-bib-0026] Griffanti, L. , Salimi‐Khorshidi, G. , Beckmann, C. F. , Auerbach, E. J. , Douaud, G. , Sexton, C. E. , … Smith, S. M. (2014). ICA‐based artefact removal and accelerated fMRI acquisition for improved resting state network imaging. NeuroImage, 95, 232–247. 24657355 10.1016/j.neuroimage.2014.03.034 PMC4154346

[hbm24524-bib-1006] Gunning‐Dixon, F. M. , & Raz, N. (2000). The cognitive correlates of white matter abnormalities in normal aging: a quantitative review. Neuropsychology, 14(2), 224. 10791862 10.1037//0894-4105.14.2.224

[hbm24524-bib-0027] Habib, R. , Nyberg, L. , & Nilsson, L. G. (2007). Cognitive and non‐cognitive factors contributing to the longitudinal identification of successful older adults in the betula study. Neuropsychology, Development, and Cognition. Section B, Aging, Neuropsychology and Cognition, 14(3), 257–273. 10.1080/13825580600582412 17453560

[hbm24524-bib-0028] Hafkemeijer, A. , van der Grond, J. , & Rombouts, S. A. (2012). Imaging the default mode network in aging and dementia. Biochimica et Biophysica Acta, 1822(3), 431–441. 21807094 10.1016/j.bbadis.2011.07.008

[hbm24524-bib-0029] Han, X. , Jovicich, J. , Salat, D. , van der Kouwe, A. , Quinn, B. , Czanner, S. , … Killiany, R. (2006). Reliability of MRI‐derived measurements of human cerebral cortical thickness: The effects of field strength, scanner upgrade and manufacturer. NeuroImage, 32(1), 180–194. 16651008 10.1016/j.neuroimage.2006.02.051

[hbm24524-bib-0030] Hanggi, J. , Langer, N. , Lutz, K. , Birrer, K. , Merillat, S. , & Jancke, L. (2015). Structural brain correlates associated with professional handball playing. PLoS One, 10(4), e0124222 10.1371/journal.pone.0124222 25915906 PMC4411074

[hbm24524-bib-0031] Harada, C. N. , Love, M. C. N. , & Triebel, K. L. (2013). Normal cognitive aging. Clinics in Geriatric Medicine, 29(4), 737–752. 24094294 10.1016/j.cger.2013.07.002 PMC4015335

[hbm24524-bib-0032] Hartshorne, J. K. , & Germine, L. T. (2015). When does cognitive functioning peak? The asynchronous rise and fall of different cognitive abilities across the life span. Psychological Science, 26(4), 433–443. 10.1177/0956797614567339 25770099 PMC4441622

[hbm24524-bib-0033] Hedden, T. , & Gabrieli, J. D. (2004). Insights into the ageing mind: A view from cognitive neuroscience. Nature Reviews Neuroscience, 5(2), 87–96. 14735112 10.1038/nrn1323

[hbm24524-bib-0034] Hogstrom, L. J. , Westlye, L. T. , Walhovd, K. B. , & Fjell, A. M. (2013). The structure of the cerebral cortex across adult life: Age‐related patterns of surface area, thickness, and gyrification. Cerebral Cortex, 23(11), 2521–2530. 22892423 10.1093/cercor/bhs231

[hbm24524-bib-0035] Jack, C. R., Jr. , Bernstein, M. A. , Fox, N. C. , Thompson, P. , Alexander, G. , Harvey, D. , … Weiner, M. W. (2008). The Alzheimer's disease neuroimaging initiative (ADNI): MRI methods. Journal of Magnetic Resonance Imaging, 27(4), 685–691. 10.1002/jmri.21049 18302232 PMC2544629

[hbm24524-bib-0036] Jahanshad, N. , & Thompson, P. M. (2017). Multimodal neuroimaging of male and female brain structure in health and disease across the life span. Journal of Neuroscience Research, 95(1–2), 371–379. 10.1002/jnr.23919 27870421 PMC5119539

[hbm24524-bib-0037] Jancke, L. , Merillat, S. , Liem, F. , & Hanggi, J. (2015). Brain size, sex, and the aging brain. Human Brain Mapping, 36(1), 150–169. 10.1002/hbm.22619 25161056 PMC6869393

[hbm24524-bib-0038] Jockwitz, C. , Caspers, S. , Lux, S. , Jutten, K. , Schleicher, A. , Eickhoff, S. B. , … Zilles, K. (2017). Age‐ and function‐related regional changes in cortical folding of the default mode network in older adults. Brain Structure and Function, 222(1), 83–99. 10.1007/s00429-016-1202-4 26943919

[hbm24524-bib-0039] Jones, D. T. , Machulda, M. M. , Vemuri, P. , McDade, E. M. , Zeng, G. , Senjem, M. L. , … Jack, C. R. (2011). Age‐related changes in the default mode network are more advanced in Alzheimer disease. Neurology, 77(16), 1524–1531. 21975202 10.1212/WNL.0b013e318233b33d PMC3198977

[hbm24524-bib-0040] Jovicich, J. , Marizzoni, M. , Sala‐Llonch, R. , Bosch, B. , Bartres‐Faz, D. , Arnold, J. , … PharmaCog Consortium . (2013). Brain morphometry reproducibility in multi‐center 3T MRI studies: A comparison of cross‐sectional and longitudinal segmentations. NeuroImage, 83, 472–484. 10.1016/j.neuroimage.2013.05.007 23668971

[hbm24524-bib-1005] Kalbe, E. , Kessler, J. , Calabrese, P. , Smith, R. , Passmore, A. P. , Brand, M. A. , & Bullock, R. (2004). DemTect: a new, sensitive cognitive screening test to support the diagnosis of mild cognitive impairment and early dementia. International journal of geriatric psychiatry, 19(2), 136–143. 14758579 10.1002/gps.1042

[hbm24524-bib-0041] Kaup, A. R. , Mirzakhanian, H. , Jeste, D. V. , & Eyler, L. T. (2011). A review of the brain structure correlates of successful cognitive aging. The Journal of Neuropsychiatry and Clinical Neurosciences, 23(1), 6–15. 10.1176/appi.neuropsych.23.1.6 10.1176/appi.neuropsych.23.1.6 21304134 PMC3068909

[hbm24524-bib-0042] Kohncke, Y. , Laukka, E. J. , Brehmer, Y. , Kalpouzos, G. , Li, T. Q. , Fratiglioni, L. , … Lovden, M. (2016). Three‐year changes in leisure activities are associated with concurrent changes in white matter microstructure and perceptual speed in individuals aged 80 years and older. Neurobiology of Aging, 41, 173–186. 10.1016/j.neurobiolaging.2016.02.013 27103530

[hbm24524-bib-0043] Kruggel, F. , Turner, J. , Muftuler, L. T. , & Alzheimer's Disease Neuroimaging, I . (2010). Impact of scanner hardware and imaging protocol on image quality and compartment volume precision in the ADNI cohort. NeuroImage, 49(3), 2123–2133. 10.1016/j.neuroimage.2009.11.006 19913626 PMC2951115

[hbm24524-bib-0044] Laukka, E. J. , Lovden, M. , Herlitz, A. , Karlsson, S. , Ferencz, B. , Pantzar, A. , … Backman, L. (2013). Genetic effects on old‐age cognitive functioning: A population‐based study. Psychology and Aging, 28(1), 262–274. 10.1037/a0030829 23276211

[hbm24524-bib-0045] Lehrl, S. (2005). In Verlag S. (Ed.), Mehrfachwahl‐Wortschatz‐Intelligenztest MWT‐B. Balingen, Germany: 5. Unveränderte Auflage.

[hbm24524-bib-0046] Lemaitre, H. , Goldman, A. L. , Sambataro, F. , Verchinski, B. A. , Meyer‐Lindenberg, A. , Weinberger, D. R. , & Mattay, V. S. (2012). Normal age‐related brain morphometric changes: Nonuniformity across cortical thickness, surface area and gray matter volume? Neurobiology of Aging, 33(3), 617 e611–619. 10.1016/j.neurobiolaging.2010.07.013 PMC3026893 20739099

[hbm24524-bib-0047] Liem, F. , Merillat, S. , Bezzola, L. , Hirsiger, S. , Philipp, M. , Madhyastha, T. , & Jancke, L. (2015). Reliability and statistical power analysis of cortical and subcortical FreeSurfer metrics in a large sample of healthy elderly. NeuroImage, 108, 95–109. 10.1016/j.neuroimage.2014.12.035 25534113

[hbm24524-bib-0048] Liu, T. , Wen, W. , Zhu, W. , Kochan, N. A. , Trollor, J. N. , Reppermund, S. , … Sachdev, P. S. (2011). The relationship between cortical sulcal variability and cognitive performance in the elderly. NeuroImage, 56(3), 865–873. 21397704 10.1016/j.neuroimage.2011.03.015

[hbm24524-bib-0049] Long, X. , Liao, W. , Jiang, C. , Liang, D. , Qiu, B. , & Zhang, L. (2012). Healthy aging: An automatic analysis of global and regional morphological alterations of human brain. Academic Radiology, 19(7), 785–793. 10.1016/j.acra.2012.03.006 22503890

[hbm24524-bib-0050] Lovden, M. , Karalija, N. , Andersson, M. , Wahlin, A. , Jan, A. , Kohncke, Y. , … Lindenberger, U. (2017). Latent‐profile analysis reveals behavioral and brain correlates of dopamine‐cognition associations. Cerebral Cortex, 28, 1–14. 10.1093/cercor/bhx253 PMC5823239 29028935

[hbm24524-bib-0051] Miller, D. I. , Taler, V. , Davidson, P. S. , & Messier, C. (2012). Measuring the impact of exercise on cognitive aging: Methodological issues. Neurobiology of Aging, 33(3), 622 e629–643–622.e43. 10.1016/j.neurobiolaging.2011.02.020 21514694

[hbm24524-bib-0052] Miller, K. L. , Alfaro‐Almagro, F. , Bangerter, N. K. , Thomas, D. L. , Yacoub, E. , Xu, J. , … Smith, S. M. (2016). Multimodal population brain imaging in the UK Biobank prospective epidemiological study. Nature Neuroscience, 19(11), 1523–1536. 10.1038/nn.4393 27643430 PMC5086094

[hbm24524-bib-0053] Morris, J. C. , Heyman, A. , Mohs, R. C. , Hughes, J. P. , Van Belle, G. , Fillenbaum, G. , … Clark, C. (1989). The consortium to establish a registry for Alzheimer's disease (CERAD). Part I. Clinical and neuropsychological assessment of Alzheimer's disease. Neurology, 39(9), 1159–1165. 2771064 10.1212/wnl.39.9.1159

[hbm24524-bib-0054] O'Sullivan, M. , Jones, D. K. , Summers, P. E. , Morris, R. G. , Williams, S. C. , & Markus, H. S. (2001). Evidence for cortical "disconnection" as a mechanism of age‐related cognitive decline. Neurology, 57(4), 632–638. 11524471 10.1212/wnl.57.4.632

[hbm24524-bib-0055] Park, D. C. , & Reuter‐Lorenz, P. (2009). The adaptive brain: Aging and neurocognitive scaffolding. Annual Review of Psychology, 60, 173–196. 10.1146/annurev.psych.59.103006.093656 PMC3359129 19035823

[hbm24524-bib-0056] Raz, N. , Lindenberger, U. , Rodrigue, K. M. , Kennedy, K. M. , Head, D. , Williamson, A. , … Acker, J. D. (2005). Regional brain changes in aging healthy adults: General trends, individual differences and modifiers. Cerebral Cortex, 15(11), 1676–1689. 10.1093/cercor/bhi044 15703252

[hbm24524-bib-0057] Raz, N. , & Rodrigue, K. M. (2006). Differential aging of the brain: Patterns, cognitive correlates and modifiers. Neuroscience and Biobehavioral Reviews, 30(6), 730–748. 16919333 10.1016/j.neubiorev.2006.07.001 PMC6601348

[hbm24524-bib-0058] Reuter‐Lorenz, P. A. , & Cappell, K. A. (2008). Neurocognitive aging and the compensation hypothesis. Current Directions in Psychological Science, 17(3), 177–182.

[hbm24524-bib-0059] Reuter‐Lorenz, P. A. , & Lustig, C. (2005). Brain aging: Reorganizing discoveries about the aging mind. Current Opinion in Neurobiology, 15(2), 245–251. 15831410 10.1016/j.conb.2005.03.016

[hbm24524-bib-0060] Reuter‐Lorenz, P. A. , & Park, D. C. (2014). How does it STAC up? Revisiting the scaffolding theory of aging and cognition. Neuropsychology Review, 24(3), 355–370. 25143069 10.1007/s11065-014-9270-9 PMC4150993

[hbm24524-bib-0061] Richardson, J. T. E. (2011). Eta squared and partial eta squared as measures of effect size in educational research. Educational Research Review, 6(2), 135–147. 10.1016/j.edurev.2010.12.001

[hbm24524-bib-0062] Ritchie, S. J. , Bates, T. C. , Der, G. , Starr, J. M. , & Deary, I. J. (2013). Education is associated with higher later life IQ scores, but not with faster cognitive processing speed. Psychology and Aging, 28(2), 515–521. 10.1037/a0030820 23276218

[hbm24524-bib-0063] Salat, D. H. , Buckner, R. L. , Snyder, A. Z. , Greve, D. N. , Desikan, R. S. , Busa, E. , … Fischl, B. (2004). Thinning of the cerebral cortex in aging. Cerebral Cortex, 14(7), 721–730. 10.1093/cercor/bhh032 15054051

[hbm24524-bib-0064] Salat, D. H. , Tuch, D. S. , Hevelone, N. D. , Fischl, B. , Corkin, S. , Rosas, H. D. , & Dale, A. M. (2005). Age‐related changes in prefrontal white matter measured by diffusion tensor imaging. Annals of the New York Academy of Sciences, 1064, 37–49. 16394146 10.1196/annals.1340.009

[hbm24524-bib-0065] Salimi‐Khorshidi, G. , Douaud, G. , Beckmann, C. F. , Glasser, M. F. , Griffanti, L. , & Smith, S. M. (2014). Automatic denoising of functional MRI data: Combining independent component analysis and hierarchical fusion of classifiers. NeuroImage, 90, 449–468. 24389422 10.1016/j.neuroimage.2013.11.046 PMC4019210

[hbm24524-bib-0066] Salthouse, T. A. (2010). Selective review of cognitive aging. Journal of the International Neuropsychological Society, 16(5), 754–760. 20673381 10.1017/S1355617710000706 PMC3637655

[hbm24524-bib-0067] Schaie, K. W. (1993). The Seattle longitudinal study: A thirty‐five‐year inquiry of adult intellectual development. Zeitschrift für Gerontologie, 26(3), 129–137. 8337905

[hbm24524-bib-0068] Schaie, K. W. , & Willis, S. L. (2010). The Seattle longitudinal study of adult cognitive development. ISSBD Bulletin, 57(1), 24–29. 23536918 PMC3607395

[hbm24524-bib-0069] Schaie, K. W. , Willis, S. L. , & Caskie, G. I. (2004). The Seattle longitudinal study: Relationship between personality and cognition. Neuropsychology, Development, and Cognition. Section B, Aging, Neuropsychology and Cognition, 11(2–3), 304–324. 10.1080/13825580490511134 PMC1474018 16755303

[hbm24524-bib-0070] Schlett, C. L. , Hendel, T. , Hirsch, J. , Weckbach, S. , Caspers, S. , Schulz‐Menger, J. , … Bamberg, F. (2016). Quantitative, organ‐specific interscanner and intrascanner variability for 3T whole‐body magnetic resonance imaging in a multicenter, multivendor study. Investigative Radiology, 51(4), 255–265. 10.1097/RLI.0000000000000237 26646309

[hbm24524-bib-1004] Schmidt, K.‐H. , & Metzler, P. (1992). Wortschatztest: WST: Beltz.

[hbm24524-bib-0071] Schmermund, A. , Möhlenkamp, S. , Stang, A. , Grönemeyer, D. , Seibel, R. , Hirche, H. , … Erbel, R. (2002). Assessment of clinically silent atherosclerotic disease and established and novel risk factors for predicting myocardial infarction and cardiac death in healthy middle‐aged subjects: Rationale and design of the Heinz Nixdorf RECALL study. American Heart Journal, 144(2), 212–218. 10.1067/mhj.2002.123579 12177636

[hbm24524-bib-0072] Schneeweis, N. , Skirbekk, V. , & Winter‐Ebmer, R. (2014). Does education improve cognitive performance four decades after school completion? Demography, 51(2), 619–643. 10.1007/s13524-014-0281-1 24578168

[hbm24524-bib-1002] Silberzahn, R. , Uhlmann, E. L. , Martin, D. P. , Anselmi, P. , Aust, F. , Awtrey, E. , … Carlsson, R. (2018). Many analysts, one data set: Making transparent how variations in analytic choices affect results. Advances in Methods and Practices in Psychological Science, 1(3), 337–356.

[hbm24524-bib-0073] Sofi, F. , Valecchi, D. , Bacci, D. , Abbate, R. , Gensini, G. F. , Casini, A. , & Macchi, C. (2011). Physical activity and risk of cognitive decline: A meta‐analysis of prospective studies. Journal of Internal Medicine, 269(1), 107–117. 10.1111/j.1365-2796.2010.02281.x 20831630

[hbm24524-bib-0074] Sowell, E. R. , Peterson, B. S. , Thompson, P. M. , Welcome, S. E. , Henkenius, A. L. , & Toga, A. W. (2003). Mapping cortical change across the human life span. Nature Neuroscience, 6(3), 309–315. 12548289 10.1038/nn1008

[hbm24524-bib-0075] Stonnington, C. M. , Tan, G. , Kloppel, S. , Chu, C. , Draganski, B. , Jack, C. R., Jr. , … Frackowiak, R. S. (2008). Interpreting scan data acquired from multiple scanners: A study with Alzheimer's disease. NeuroImage, 39(3), 1180–1185. 10.1016/j.neuroimage.2007.09.066 18032068 PMC2225446

[hbm24524-bib-1001] Sturm, W. , Willmes, K. , & Horn, W. (1993). Leistungsprüfsystem für 50‐ bis 90‐Jährige (LPS 50+) (2nd ed.). Göttingen: Hogrefe.

[hbm24524-bib-0076] Sudlow, C. , Gallacher, J. , Allen, N. , Beral, V. , Burton, P. , Danesh, J. , … Collins, R. (2015). UK Biobank: An open access resource for identifying the causes of a wide range of complex diseases of middle and old age. PLoS Medicine, 12(3), e1001779 10.1371/journal.pmed.1001779 25826379 PMC4380465

[hbm24524-bib-0077] Thompson, P. M. , Stein, J. L. , Medland, S. E. , Hibar, D. P. , Vasquez, A. A. , Renteria, M. E. , … Saguenay Youth Study (SYS) Group . (2014). The ENIGMA consortium: Large‐scale collaborative analyses of neuroimaging and genetic data. Brain Imaging and Behavior, 8(2), 153–182. 10.1007/s11682-013-9269-5 24399358 PMC4008818

[hbm24524-bib-0078] Trachtenberg, A. J. , Filippini, N. , Ebmeier, K. P. , Smith, S. M. , Karpe, F. , & Mackay, C. E. (2012). The effects of APOE on the functional architecture of the resting brain. NeuroImage, 59(1), 565–572. 10.1016/j.neuroimage.2011.07.059 21851856

[hbm24524-bib-0079] Tucker‐Drob, E. M. , Johnson, K. E. , & Jones, R. N. (2009). The cognitive reserve hypothesis: A longitudinal examination of age‐associated declines in reasoning and processing speed. Developmental Psychology, 45(2), 431–446. 10.1037/a0014012 19271829 PMC3230274

[hbm24524-bib-0080] van Hooren, S. A. , Valentijn, A. M. , Bosma, H. , Ponds, R. W. , van Boxtel, M. P. , & Jolles, J. (2007). Cognitive functioning in healthy older adults aged 64‐81: A cohort study into the effects of age, sex, and education. Neuropsychology, Development, and Cognition. Section B, Aging, Neuropsychology and Cognition, 14(1), 40–54. 10.1080/138255890969483 17164189

[hbm24524-bib-0081] Walhovd, K. B. , Fjell, A. M. , Westerhausen, R. , Nyberg, L. , Ebmeier, K. P. , Lindenberger, U. , … Lifebrain, C. (2018). Healthy minds 0‐100 years: Optimising the use of European brain imaging cohorts ("Lifebrain"). European Psychiatry, 50, 47–56. 10.1016/j.eurpsy.2017.12.006 29449073

[hbm24524-bib-0082] Walhovd, K. B. , Westlye, L. T. , Amlien, I. , Espeseth, T. , Reinvang, I. , Raz, N. , … Fjell, A. M. (2011). Consistent neuroanatomical age‐related volume differences across multiple samples. Neurobiology of Aging, 32(5), 916–932. 10.1016/j.neurobiolaging.2009.05.013 19570593 PMC4040218

[hbm24524-bib-1003] Ware, J. E., Jr. , Kosinski, M. , & Keller, S. D. (1996). A 12‐Item Short‐Form Health Survey: construction of scales and preliminary tests of reliability and validity. Medical care, 34(3), 220–233. 8628042 10.1097/00005650-199603000-00003

[hbm24524-bib-0083] Westlye, L. T. , Walhovd, K. B. , Dale, A. M. , Espeseth, T. , Reinvang, I. , Raz, N. , … Fjell, A. M. (2009). Increased sensitivity to effects of normal aging and Alzheimer's disease on cortical thickness by adjustment for local variability in gray/white contrast: A multi‐sample MRI study. NeuroImage, 47(4), 1545–1557. 10.1016/j.neuroimage.2009.05.084 19501655 PMC2828679

[hbm24524-bib-0084] Zahodne, L. B. , Glymour, M. M. , Sparks, C. , Bontempo, D. , Dixon, R. A. , MacDonald, S. W. , & Manly, J. J. (2011). Education does not slow cognitive decline with aging: 12‐year evidence from the Victoria longitudinal study. Journal of the International Neuropsychological Society, 17(6), 1039–1046. 10.1017/S1355617711001044 21923980 PMC3285821

[hbm24524-bib-0085] Ziegler, G. , Dahnke, R. , Gaser, C. , & Alzheimer's Disease Neuroimaging, I . (2012). Models of the aging brain structure and individual decline. Frontiers in Neuroinformatics, 6, 3 10.3389/fninf.2012.00003 22435060 PMC3303090

[hbm24524-bib-0086] Ziegler, G. , Dahnke, R. , Jancke, L. , Yotter, R. A. , May, A. , & Gaser, C. (2012). Brain structural trajectories over the adult lifespan. Human Brain Mapping, 33(10), 2377–2389. 10.1002/hbm.21374 21898677 PMC6870331

[hbm24524-bib-0087] Zollig, J. , Merillat, S. , Eschen, A. , Rocke, C. , Martin, M. , & Jancke, L. (2011). Plasticity and imaging research in healthy aging: Core ideas and profile of the International Normal Aging and Plasticity Imaging Center (INAPIC). Gerontology, 57(2), 190–192. 10.1159/000324307 21307637

