# Supplementary material for: Generalizing age effects on brain structure and cognition: A two‐study comparison approach
Source: Hum Brain Mapp. 2019 Jan 22;40(8):2305–19. doi: 10.1002/hbm.24524 (PMC6590363; doi:10.1002/hbm.24524)
Supplement: Supplementary file 1 — Table S1 Overview of general linear models assessing age‐related differences in cognitive abilities for each sample individually (1000BRAINS/LHAB/pooled analysis): Results presented for the three different models: BASE, MAIN, and SENS, with F‐values (p‐values/partial eta square). Significant results (p < 0.05) are written in bold; significant results even after correcting for multiple comparisons for the two samples 1000BRAINS and LHAB (five cognitive tests × 2 samples × three models [p = 0.05/30]) are marked in yellow. Table S2: Overview of general linear models assessing age‐related differences in mean cortical thickness of the left and right hemisphere for each sample individually (1000BRAINS/LHAB/pooled analysis): Results presented for the three different models: BASE, MAIN, and SENS, with F‐values (p‐values/partial eta square). Significant results (p < 0.05) are written in bold; significant results even after correcting for multiple comparisons for the two samples 1000BRAINS and LHAB (two hemispheres × two samples × three models [p = 0.05/12]) are marked in yellow. Table S3: Overview of general linear models assessing age‐related differences in mean cortical thickness of the different parts of the default mode network for each sample individually (1000BRAINS/LHAB/pooled analysis): Results presented for the three different models: BASE, MAIN, and SENS, with F‐values (p‐values/partial eta square). Significant results (p < 0.05) are written in bold; significant results even after correcting for multiple comparisons for the two samples 1000BRAINS and LHAB (six regions of interest × two samples × three models [p = 0.05/36]) are marked in yellow. Table S4: Overview of general linear models assessing the relation between cognitive performance and mean cortical thickness of the different parts of the default mode network for each sample individually (1000BRAINS/LHAB/pooled analysis): Results presented for the three different models: BASE, MAIN, and SENS, with F‐values [file HBM-40-2305-s001.docx]

Table S1: Overview of General Linear Models assessing age-related differences in cognitive abilities for each sample individually (1000BRAINS / LHAB / pooled analysis): Results presented for the three different models: BASE, MAIN, and SENS, with F-values (p-values/partial eta square). Significant results (p < .05) are written in bold; significant results even after correcting for multiple comparisons for the two samples 1000BRAINS and LHAB (5 cognitive tests x 2 samples x 3 models [p = .05 / 30]) are marked in yellow.

|  | | 1000BRAINS | | | LHAB | | | POOLED | | |
| --- | --- | --- | --- | --- | --- | --- | --- | --- | --- | --- |
|  |  | BASE | MAIN | SENS | BASE | MAIN | SENS | BASE | MAIN | SENS |
| A  G  E | **Vocabulary** | 2.729 (.100/ .012) | .064 (.801/ <.001) | .09 (.764/ <.001) | .066 (.797/ <.001) | .152 (.697/ .001) | .442 (.507/ .002) | 1.78 (.183/ .004) | .357 (.551/ .001) | <.001 (.993/ <.001) |
|  | **Processing Speed** | **23.62 (<.001/ .095)** | **19.13 (<.001/ .079)** | **15.77 (<.001/ .066)** | **21.03 (<.001/ .085)** | **2.835 (<.001/ .085)** | **2.487 (<.001/ .084)** | **27.474 (<.001/ .057)** | **25.769 (<.001/ .054)** | **23.052 (<.001/ .049)** |
|  | **Concept Shifting** | **13.72 (<.001/ .057)** | **9.455 (.002/ .041)** | **7.621 (.006/ .033)** | **16.79 (<.001/ .069)** | **16.68 (<.001/ .069)** | **15.58 (<.001/ .066)** | **2.387 (<.001/ .043)** | **17.826 (<.001/ .038)** | **16.142 (<.001/ .035)** |
|  | **Reasoning** | **19.83 (<.001/ .081)** | **13.07 (<.001/ .055)** | **9.918 (.002/ .043)** | **29.85 (<.001/ .117)** | **3.364 (<.001/ .119)** | **28.83 (<.001/ .115)** | **28.686 (<.001/ .06)** | **24.018 (<.001/ .051)** | **19.045 (<.001/ .041)** |
|  | **Verbal fluency** | **8.088 (.005/ .035)** | **4.798 (.03/ .021)** | **4.752 (.03/ .021)** | **18.46 (<.001/ .076)** | **18.51 (<.001/ .076)** | **17.29 (<.001/ .072)** | **19.888 (<.001/ .042)** | **16.7 (<.001/ .036)** | **16.038 (<.001/ .034)** |
| G  E  N  D  E  R | **Vocabulary** | 1.225 (.27/ .005) | .97 (.326/ .004) | 2.548 (.112/ .011) | 2.666 (.104/ .012) | .32 (.572/ .001) | .089 (.766/ <.001) | .261 (.61/ .001) | 1.582 (.209/ .003) | 3.941 (.048/ .009) |
|  | **Processing Speed** | .853 (.357/ .004) | .075 (.784/ <.001) | .222 (.638/ .001) | .556 (.457/ .002) | .356 (.551/ .002) | .358 (.55/ .002) | 1.686 (.195/ .004) | .912 (.34/ .002) | .400 (.528/ .001) |
|  | **Concept Shifting** | .22 (.64/ .001) | .195 (.659/ .001) | .846 (.359/ .004) | 1.557 (.213/ .007) | .538 (.464/ .002) | .414 (.521/ .002) | 1.086 (.298/ .002) | .124 (.724/ <.001) | .029 (.864/ <.001) |
|  | **Reasoning** | .529 (.468/ .002) | .542 (.462/ .002) | 1.641 (.202/ .007) | **7.011 (.009/ .03)** | 3.696 (.056/ .016) | 3.075 (.081/ .014) | .06 (.807/ <.001) | 1.269 (.261/ .003) | 3.164 (.076/ .007) |
|  | **Verbal fluency** | <.001 (.988/ <.001) | .849 (.358/ .004) | .781 (.378/ .004) | 2.682 (.103/ .012) | .981 (.323/ .004) | .756 (.385/ .003) | .788 (.375/ .002) | .002 (.96/ <.001) | .011 (.915/ <.001) |
| E  D  U  C  A  T  I  O  N | **Vocabulary** | - | **81.86 (<.001/ .268)** | **66.88 (<.001/ .232)** | - | **27.26 (<.001/ .108)** | **26.85 (<.001/ .108)** | **-** | **6.178 (<.001/ .118)** | **5.174 (<.001/ .101)** |
|  | **Processing Speed** | - | **6.532 (.011/ .028)** | 1.936 (.165/ .009) | - | .369 (.544/ .002) | .384 (.536/ .002) | **-** | 1.807 (.18/ .004) | .935 (.334/ .002) |
|  | **Concept Shifting** | - | **13.53 (<.001/ .057)** | **8.01 (.005/ .035)** | - | **5.371 (.021/ .023)** | **5.189 (.024/ .023)** | - | **8.46 (.004/ .018)** | **7.174 (.008/ .016)** |
|  | **Reasoning** | - | **36.58 (<.001/ .14)** | **27.08 (<.001/ .109)** | - | **11.36 (.001/ .048)** | **11.32 (.001/ .049)** | **-** | **35.548 (<.001/ .073)** | **28.341 (<.001/ .059)** |
|  | **Verbal fluency** | - | **14.39 (<.001/ .06)** | **13.07 (<.001/ .056)** | - | **8.826 (.003/ .038)** | **8.622 (.004/ .037)** | **-** | **16.241 (<.001/ .035)** | **15.138 (<.001/ .033)** |
| P  H  Y  S  W  B | **Vocabulary** | - | - | **6.816 (.01/ .03)** | - | - | **6.497 (.011/ .028)** | - |  | **12.374 (<.001/ .027)** |
|  | **Processing Speed** | - | - | 3.011 (.084/ .013) | - | - | .015 (.903/ <.001) | - | **-** | 1.74 (.188/ .004) |
|  | **Concept Shifting** | - | - | 1.049 (.307/ .005) | - | - | .626 (.43/ .003) | - | - | .723 (.396/ .002) |
|  | **Reasoning** | - | - | 3.805 (.052/ .017) | - | - | 1.931 (.166/ .009) | - | **-** | **11.001 (.001/ .024)** |
|  | **Verbal fluency** | - | - | .068 (.794/ <.001) | - | - | 1.048 (.307/ .005) | - | **-** | .009 (.923/ <.001) |
| M  E  N  T  W  B | **Vocabulary** | - | - | 1.383 (.241/ .006) | - | - | .986 (.322/ .004) | - | - | **9.656 (.002/ .021)** |
|  | **Processing Speed** | - | - | **14.29 (<.001/ .06)** | - | - | .038 (.846/ <.001) | - | - | 3.471 (.063/ .008) |
|  | **Concept Shifting** | - | - | **6.58 (.011/ .029)** | - | - | **.**095 (.758/ <.001) | - | - | .681 (.41/ .002) |
|  | **Reasoning** | - | - | 2.802 (.096/ .012) | - | - | 1.711 (.192/ .008) | - | - | **6.45 (.011/ .014)** |
|  | **Verbal fluency** | - | - | .085 (.772/ <.001) | - | - | .400 (.528/ .002) | - | - | .393 (.531/ .001) |
| S  A  M  P  L  E | **Vocabulary** | - | - | - | - | - | - | **139.79 (<.001/ .236)** | **124.18 (<.001/ .216)** | **114.62 (<.001/ .203)** |
|  | **Processing Speed** | - | - | - | - | - | - | **9.735 (.002/ .021)** | **8.194 (.004/ .018)** | **6.76 (.01/ .015)** |
|  | **Concept Shifting** | - | - | - | - | - | - | .013 (.908/ <.001) | .346 (.557/ .001) | .53 (.467/ .001) |
|  | **Reasoning** | - | - | - | - | - | - | **8.054 (<.001/ .15)** | **67.063 (<.001/ .129)** | 59.496 (<.001/ .117) |
|  | **Verbal fluency** | - | - | - | - | - | - | **3.049 (<.001/ .062)** | **23.459 (<.001/ .049)** | **22.587 (<.001/ .048)** |

Table S2: Overview of General Linear Models assessing age-related differences in mean CT of the left and right hemisphere for each sample individually (1000BRAINS / LHAB / pooled analysis): Results presented for the three different models: BASE, MAIN, and SENS, with F-values (p-values/partial eta square). Significant results (p < .05) are written in bold; significant results even after correcting for multiple comparisons for the two samples 1000BRAINS and LHAB (2 hemispheres x 2 samples x 3 models [p = .05 / 12]) are marked in yellow.

|  | | 1000BRAINS | | | LHAB | | | POOLED | | |
| --- | --- | --- | --- | --- | --- | --- | --- | --- | --- | --- |
|  |  | BASE | MAIN | SENS | BASE | MAIN | SENS | BASE | MAIN | SENS |
| A  G  E | **left hemisphere** | **38.29 (<.001/.145)** | **33.24 (<.001/.129)** | **26.79 (<.001/.108)** | **22.99 (<.001/.093)** | **22.77 (<.001/.092)** | **21.28 (<.001/.087)** | **6.42 (<.001/.118)** | **56.84 (<.001/.112)** | **49.3 (<.001/.099)** |
|  | **right hemisphere** | **46.29 (<.001/.171)** | **4.15 (<.001/.152)** | **33.37 (<.001/.131)** | **3.67 (<.001/.12)** | **3.4 (<.001/.12)** | **28.69 (<.001/.114)** | **75.75 (<.001/.144)** | **71.6 (<.001/.137)** | **63.39 (<.001/.124)** |
| G  E  N  D  E  R | **left hemisphere** | 2.53 (.113/.011) | **4.16 (.043/**  **.018)** | **6.71 (.01/**  **.029)** | .3 (.587/.001) | .5 (.479/.002) | .67 (.414/.003) | 2.31 (.129/.005) | **3.87 (.05/.009)** | **5.79 (.017/.013)** |
|  | **right hemisphere** | 1.98 (.161/.009) | 3.78 (.053/.017) | **5.81 (.017/**  **.026)** | .04 (.841/<.001) | .13 (.721/.001) | .22 (.637/.001) | 1.24 (.265/.003) | 2.54 (.111/.006) | **3.94 (.048/.009)** |
| E  D  U  C  A  T  I  O  N | **left hemisphere** | - | **3.91 (.049/**  **.017)** | 2.02 (.157/.009) | - | .69 (.406/.003) | .64 (.424/.003) | - | **4.39 (.037/.01)** | 2.81 (.094/.006) |
|  | **right hemisphere** | - | **5.29 (.022/**  **.023)** | 3.43 (.065/.015) | - | .57 (.45/.003) | .54 (.464/.002) | - | **4.82 (.029/.011)** | 3.33 (.069/.007) |
| P  H  Y  S  W  B | **left hemisphere** | - | - | **8.36 (.004/.036)** | - | - | 1.08 (.301/.005) | - | - | **7.95 (.005/.017)** |
|  | **right hemisphere** | - | - | **7.34 (.007/.032)** | - | - | 1.07 (.301/.005) | - | - | **6.7 (.01/.015)** |
| M  E  N  T  W  B | **left hemisphere** | - | - | .06 (.804/<.001) | - | - | .29 (.59/.001) | - | - | .51 (.475/.001) |
|  | **right hemisphere** | - | - | .02 (.877/<.001) | - | - | .45 (.505/.002) | - | - | .25 (.617/.001) |
| S  A  M  P  L  E | **left hemisphere** | - | - | - | - | - | - | **65.99 (<.001/.127)** | **7.33 (<.001/.135)** | **77.12 (<.001/.147)** |
|  | **right hemisphere** | - | - | - | - | - | - | **43.75 (<.001/.088)** | **47.76 (<.001/.096)** | **52.68 (<.001/.105)** |

Table S3: Overview of General Linear Models assessing age-related differences in mean CT of the different parts of the DMN for each sample individually (1000BRAINS / LHAB / pooled analysis): Results presented for the three different models: BASE, MAIN, and SENS, with F-values (p-values/partial eta square). Significant results (p < .05) are written in bold; significant results even after correcting for multiple comparisons for the two samples 1000BRAINS and LHAB (6 regions of interest x 2 samples x 3 models [p = .05 / 36]) are marked in yellow.

Table S4: Overview of General Linear Models assessing the relation between cognitive performance and mean CT of the different parts of the DMN for each sample individually (1000BRAINS / LHAB / pooled analysis): Results presented for the three different models: BASE, MAIN, and SENS, with F-values (p-values/partial eta square). Significant results (p < .05) are written in bold; significant results even after correcting for multiple comparisons for the two samples 1000BRAINS and LHAB (6 regions of interest x 2 samples x 3 models [p = .05 / 36]) are marked in yellow.

|  | | 1000BRAINS | | | LHAB | | | POOLED | | |
| --- | --- | --- | --- | --- | --- | --- | --- | --- | --- | --- |
|  |  | BASE | MAIN | SENS | BASE | MAIN | SENS | BASE | MAIN | SENS |
| LEFT  aDMN | **Vocabulary** | .03 (.856/ <.001) | .01 (.913/ <.001) | .07 (.787/ <.001) | 0 (.984/ <.001) | .19 (.664/ <.001) | .39 (.532/ .002) | .02 (.881/ <.001) | .09 (.767/ <.001) | .08 (.779/ <.001) |
|  | **Processing Speed** | .83 (.364/ .004) | .79 (.374/ .004) | 1.32 (.251/ .006) | 2.45 (.119/ .011) | 2.64 (.106/ .012) | 2.58 (.11/ .012) | .11 (.741/ <.001) | .19 (.664/ <.001) | .12 (.734/ <.001) |
|  | **Concept Shifting** | .18 (.672/ <.001) | .22 (.638/ .001) | .13 (.716/ <.001) | 1.18 (.279/ .005) | 1.68 (.196/ .008) | 1.83 (.177/ .008) | 1.14 (.285/ .003) | 1.63 (.202/ .004) | 1.49 (.223/ .003) |
|  | **Reasoning** | .13 (.718/ <.001) | .21 (.649/ <.001) | .08 (.774/ <.001) | .2 (.659/ <.001) | .55 (.457/ .003) | .65 (.421/ .003) | .35 (.554/ <.001) | .84 (.361/ .002) | .77 (.382/ .002) |
|  | **Verbal fluency** | .03 (.86/ <.001) | .05 (.82/ <.001) | .05 (.82/ <.001) | .3 (.585/ .001) | .67 (.415/ .003) | .79 (.375/ .004) | .17 (.679/ <.001) | .42 (.518/ <.001) | .4 (.529/ <.001) |
| R IGHT  aDMN | **Vocabulary** | **4.64 (.032/ .021)** | 1.17 (.28/ .005) | .43 (.514/ .002) | .99 (.322/ .004) | 1.26 (.263/ .006) | 2.29 (.132/ .01) | 1.19 (.276/ .003) | .05 (.817/ <.001) | .17 (.676/ <.001) |
|  | **Processing Speed** | .03 (.864/ <.001) | .06 (.808/ <.001) | .89 (.347/ .004) | .37 (.541/ .002) | .39 (.535/ .002) | .38 (.538/ .002) | .02 (.875/ <.001) | .14 (.709/ <.001) | .69 (.406/ .002) |
|  | **Concept Shifting** | 1.71 (.192/ .008) | .62 (.433/ .003) | .17 (.676/ <.001) | .59 (.442/ .003) | .65 (.42/ .003) | .87 (.352/ .004) | .16 (.688/ <.001) | 0 (.994/ <.001) | .12 (.732/ <.001) |
|  | **Reasoning** | 0 (.962/ <.001) | .97 (.327/ .004) | 2.19 (.141/ .01) | .01 (.906/ <.001) | .01 (.938/ <.001) | .01 (.905/ <.001) | 0 (.978/ <.001) | .42 (.516/ <.001) | 1.35 (.247/ .003) |
|  | **Verbal fluency** | .07 (.787/ <.001) | .82 (.366/ .004) | .82 (.366/ .004) | .1 (.751/ <.001) | .13 (.717/ <.001) | .28 (.595/ .001) | .28 (.596/ <.001) | 1.02 (.314/ .002) | 1.24 (.265/ .003) |
| LEFT  MED I AL  pDMN | **Vocabulary** | .38 (.536/ .002) | .3 (.584/ .001) | .45 (.505/ .002) | 1.35 (.246/ .006) | 2.22 (.138/ .01) | 1.39 (.239/ .006) | .02 (.875/ <.001) | .34 (.561/ <.001) | .09 (.768/ <.001) |
|  | **Processing Speed** | .23 (.635/ .001) | .19 (.666/ <.001) | .17 (.685/ <.001) | .01 (.933/ <.001) | 0 (.964/ <.001) | 0 (.959/ <.001) | .15 (.696/ <.001) | .09 (.764/ <.001) | .17 (.683/ <.001) |
|  | **Concept Shifting** | **3.92 (.049/ .018)** | 3.83 (.051/ .017) | 3.67 (.057/ .017) | .95 (.33/ .004) | .76 (.385/ .003) | .95 (.332/ .004) | **4.73 (.03/ .01)** | **4.17 (.042/ .009)** | **4.58 (.033/ .01)** |
|  | **Reasoning** | 1.93 (.166/ .009) | 1.89 (.17/ .009) | 2.13 (.146/ .01) | .05 (.822/ <.001) | 0 (.947/ <.001) | .06 (.809/ <.001) | 1.59 (.208/ .004) | 1.1 (.294/ .002) | 1.56 (.212/ .004) |
|  | **Verbal fluency** | .17 (.683/ <.001) | .25 (.619/ .001) | .29 (.593/ .001) | .14 (.713/ <.001) | .05 (.82/ <.001) | .14 (.709/ <.001) | 0 (.958/ <.001) | .06 (.802/ <.001) | .04 (.835/ <.001) |
| R IGHT  MED  pDMN | **Vocabulary** | .58 (.448/ .003) | .48 (.491/ .002) | .18 (.676/ <.001) | .41 (.521/ .002) | .43 (.511/ .002) | 1.09 (.297/ .005) | .05 (.817/ <.001) | 0 (.957/ <.001) | .21 (.646/ <.001) |
|  | **Processing Speed** | .6 (.438/ .003) | .53 (.466/ .002) | .11 (.746/ <.001) | .13 (.717/ <.001) | .13 (.714/ <.001) | .13 (.723/ <.001) | .62 (.433/ .001) | .55 (.458/ .001) | .15 (.695/ <.001) |
|  | **Concept Shifting** | .88 (.35/ .004) | 1.06 (.305/ .005) | 1.59 (.208/ .007) | 2.08 (.151/ .009) | 2.09 (.15/ .009) | 2.44 (.12/ .011) | 2.95 (.086/ .007) | 3.36 (.068/ .007) | **4.4 (.037/ .01)** |
|  | **Reasoning** | 1.6 (.208/ .007) | 2.19 (.141/ .01) | 3.27 (.072/ .015) | .07 (.796/ <.001) | .06 (.803/ <.001) | .21 (.649/ <.001) | 1.54 (.215/ .003) | 2.03 (.155/ .005) | 3.35 (.068/ .007) |
|  | **Verbal fluency** | .08 (.771/ <.001) | .15 (.7/ <.001) | .15 (.697/ <.001) | .74 (.392/ .003) | .74 (.391/ .003) | 1.06 (.305/ .005) | .92 (.339/ .002) | 1.16 (.282/ .003) | 1.35 (.246/ .003) |
| LEFT  LAT  pDMN | **Vocabulary** | 2.94 (.088/ .013) | 1.41 (.237/ .006) | 1.24 (.267/ .006) | 3.81 (.052/ .017) | **4.98 (.027/ .022)** | **5.81 (.017/ .026)** | .19 (.663/ <.001) | .1 (.753/ <.001) | .29 (.59/ <.001) |
|  | **Processing Speed** | 1.51 (.22/ .007) | 1.01 (.316/ .005) | .73 (.395/ .003) | .01 (.914/ <.001) | .01 (.933/ <.001) | .01 (.94/ <.001) | .9 (.342/ .002) | .6 (.44/ .001) | .29 (.59/ <.001) |
|  | **Concept Shifting** | .01 (.909/ <.001) | .03 (.852/ <.001) | .09 (.764/ <.001) | .34 (.56/ .002) | .44 (.508/ .002) | .5 (.48/ .002) | .01 (.918/ <.001) | .19 (.666/ <.001) | .36 (.546/ <.001) |
|  | **Reasoning** | **6.59 (.011/ .029)** | **4.87 (.028/ .022)** | **4.52 (.035/ .02)** | 1.31 (.254/ .006) | 1.14 (.287/ .005) | .9 (.345/ .004) | **8.07 (.005/ .018)** | **5.97 (.015/ .013)** | **5.09 (.025/ .011)** |
|  | **Verbal fluency** | .12 (.729/ <.001) | .51 (.475/ .002) | .53 (.469/ .002) | .18 (.669/ <.001) | .28 (.597/ .001) | .37 (.545/ .002) | .23 (.629/ <.001) | .77 (.38/ .002) | .86 (.354/ .002) |
| R IGHT  LAT  pDMN | **Vocabulary** | .1 (.747/ <.001) | .54 (.463/ .002) | .39 (.534/ .002) | 1.41 (.237/ .006) | .94 (.333/ .004) | .91 (.342/ .004) | .58 (.446/ .001) | .74 (.389/ .002) | .65 (.42/ .001) |
|  | **Processing Speed** | .4 (.526/ .002) | .57 (.451/ .003) | .37 (.546/ .002) | .81 (.368/ .004) | .89 (.347/ .004) | .86 (.354/ .004) | .01 (.931/ <.001) | .01 (.934/ <.001) | 0 (.963/ <.001) |
|  | **Concept Shifting** | 2.08 (.15/ .009) | 2.62 (.107/ .012) | 2.37 (.125/ .011) | 2.59 (.109/ .012) | 2.24 (.136/ .01) | 2.17 (.142/ .01) | **4.17 (.042/ .009)** | **4.35 (.038/ .01)** | **4.44 (.036/ .01)** |
|  | **Reasoning** | 1.78 (.183/ .008) | 1.39 (.24/ .006) | 1.77 (.185/ .008) | .94 (.333/ .004) | 1.39 (.241/ .006) | 1.32 (.251/ .006) | 3.23 (.073/ .007) | 3.47 (.063/ .008) | 3.61 (.058/ .008) |
|  | **Verbal fluency** | .55 (.459/ .003) | .87 (.351/ .004) | .86 (.355/ .004) | .22 (.637/ .001) | .42 (.519/ .002) | .42 (.518/ .002) | .02 (.901/ <.001) | .02 (.891/ <.001) | .02 (.893/ <.001) |
| AGE | **Vocabulary** | .87 (.351/ .004) | .03 (.863/ <.001) | .32 (.574/ .001) | .79 (.375/ .004) | .95 (.33/ .004) | 1.55 (.214/ .007) | .12 (.724/ <.001) | .23 (.632/ <.001) | 1.01 (.316/ .002) |
|  | **Processing Speed** | **17.21 (<.001/ .073)** | **14.28 (<.001/ .061)** | **11.58 (<.001/ .051)** | **22.79 (<.001/ .094)** | **22.7 (<.001/ .094)** | **22.17 (<.001/ .093)** | **41.2 (<.001/ .085)** | **38.79 (<.001/ .08)** | **35.69 (<.001/ .075)** |
|  | **Concept Shifting** | **8.85 (.003/ .039)** | **6.36 (.012/ .028)** | **5.2 (.024/ .024)** | **9.36 (.002/ .041)** | **9.47 (.002/ .042)** | **8.72 (.004/ .039)** | **19.82 (<.001/ .043)** | **17.27 (<.001/ .037)** | **15.33 (<.001/ .033)** |
|  | **Reasoning** | **14.68 (<.001/ .063)** | **1.3 (.002/ .045)** | **7.91 (.005/ .035)** | **23.75 (<.001/ .098)** | **24.72 (<.001/ .102)** | **23.42 (<.001/ .098)** | **38.22 (<.001/ .079)** | **34.45 (<.001/ .072)** | **3.2 (<.001/ .064)** |
|  | **Verbal fluency** | **4.39 (.037/ .02)** | 2.45 (.119/ .011) | 2.41 (.122/ .011) | **15.18 (<.001/ .065)** | **15.61 (<.001/ .067)** | **14.49 (<.001/ .063)** | **18.58 (<.001/ .04)** | **15.83 (<.001/ .034)** | **14.82 (<.001/ .032)** |
| GENDER | **Vocabulary** | .75 (.389/ .003) | .94 (.332/ .004) | 2.17 (.142/ .01) | 2.85 (.093/ .013) | .43 (.511/ .002) | .12 (.73/ <.001) | 3.74 (.054/ .008) | .02 (.879/ <.001) | .64 (.424/ .001) |
|  | **Processing Speed** | .86 (.354/ .004) | .1 (.748/ <.001) | .16 (.685/ <.001) | .74 (.391/ .003) | .46 (.498/ .002) | .44 (.506/ .002) | 1.33 (.249/ .003) | .4 (.529/ <.001) | .04 (.851/ <.001) |
|  | **Concept Shifting** | .01 (.943/ <.001) | .48 (.491/ .002) | 1.12 (.29/ .005) | 1.56 (.213/ .007) | .56 (.455/ .003) | .4 (.528/ .002) | 1.27 (.261/ .003) | .04 (.846/ <.001) | .02 (.883/ <.001) |
|  | **Reasoning** | 1.02 (.314/ .005) | .08 (.779/ <.001) | .71 (.4/ .003) | **6.35 (.012/ .028)** | 3.32 (.07/ .015) | 2.69 (.102/ .012) | **5.83 (.016/ .013)** | 1.04 (.309/ .002) | .26 (.611/ <.001) |
| EDUCAT I ON | **Verbal fluency** | .03 (.861/ <.001) | .53 (.469/ .002) | .5 (.479/ .002) | 2.54 (.112/ .011) | .93 (.337/ .004) | .66 (.419/ .003) | 1.46 (.227/ .003) | .02 (.899/ <.001) | 0 (.995/ <.001) |
|  | **Vocabulary** | - | **69.99 (<.001/ .243)** | **6.82 (<.001/ .22)** | - | **28.64 (<.001/ .116)** | **28.33 (<.001/ .116)** | - | **98.39 (<.001/ .181)** | **89.59 (<.001/ .168)** |
|  | **Processing Speed** | - | **6.55 (.011/ .029)** | 2.52 (.114/ .012) | - | .62 (.432/ .003) | .62 (.434/ .003) | - | **4.94 (.027/ .011)** | 3.14 (.077/ .007) |
|  | **Concept Shifting** | - | **1.82 (.001/ .047)** | **7.1 (.008/ .032)** | - | **5.67 (.018/ .025)** | 5.45 (.02/ .025) | - | **16.76 (<.001/ .036)** | **14 (<.001/ .031)** |
|  | **Reasoning** | - | **33.15 (<.001/ .132)** | **26.02 (<.001/ .107)** | - | **11.84 (<.001/ .052)** | **11.68 (<.001/ .051)** | - | **4.8 (<.001/ .084)** | **35.37 (<.001/ .074)** |
| PHYS  WB | **Verbal fluency** | - | **15.15 (<.001/ .065)** | **13.76 (<.001/ .06)** | - | **9.39 (.002/ .041)** | **9.11 (.003/ .04)** | - | **22.99 (<.001/ .049)** | **21.48 (<.001/ .046)** |
|  | **Vocabulary** | - | - | **5.26 (.023/ .024)** | - | - | **7.45 (.007/ .033)** | - | - | **12.48 (<.001/ .027)** |
|  | **Processing Speed** | - | - | **4.31 (.039/ .02)** | - | - | 0 (.978/ <.001) | - | - | 2.26 (.134/ .005) |
|  | **Concept Shifting** | - | - | .73 (.394/ .003) | - | - | .89 (.346/ .004) | - | - | 1.71 (.191/ .004) |
|  | **Reasoning** | - | - | **4.76 (.03/ .022)** | - | - | 1.9 (.17/ .009) | - | - | **5.84 (.016/ .013)** |
| MENT  WB | **Verbal fluency** | - | - | .06 (.809/ <.001) | - | - | 1.52 (.22/ .007) | - | - | .28 (.597/ <.001) |
|  | **Vocabulary** | - | - | .96 (.328/ .004) | - | - | 1.26 (.262/ .006) | - | - | 3 (.084/ .007) |
|  | **Processing Speed** | - | - | **14.92 (<.001/ .065)** | - | - | .01 (.917/ <.001) | - | - | **9.59 (.002/ .021)** |
|  | **Concept Shifting** | - | - | **5.69 (.018/ .026)** | - | - | .11 (.74/ <.001) | - | - | **4.25 (.04/ .01)** |
|  | **Reasoning** | - | - | 3.2 (.075/ .015) | - | - | 1.26 (.263/ .006) | - | - | **4.26 (.04/ .01)** |
|  | **Verbal fluency** | - | - | .24 (.625/ .001) | - | - | .39 (.531/ .002) | - | - | .33 (.566/ <.001) |
| SAMPL E | **Vocabulary** | - | - | - | - | - | - | .37 (.542/ <.001) | 1.22 (.27/ .003) | 3.27 (.071/ .007) |
|  | **Processing Speed** | - | - | - | - | - | - | .02 (.879/ <.001) | .06 (.806/ <.001) | .5 (.48/ .001) |
|  | **Concept Shifting** | - | - | - | - | - | - | 1.58 (.21/ .004) | .28 (.597/ <.001) | .03 (.87/ <.001) |
|  | **Reasoning** | - | - | - | - | - | - | 1.46 (.228/ .003) | .01 (.918/ <.001) | .19 (.66/ <.001) |
|  | **Verbal fluency** | - | - | - | - | - | - | .03 (.866/ <.001) | .47 (.494/ .001) | .64 (.424/ .001) |

Table S5: Relation between age^2^ and cognitive performance (SENS + age^2^) with T-values and p-values in brackets.

|  | 1000BRAINS | LHAB | Pooled |
| --- | --- | --- | --- |
| vocabulary | (2.297/ 0.131) | (0.232/ 0.631) | (1.81/ 0.179) |
| processing speed | (3.094/ 0.08) | (1.682/ 0.196) | (0.027/ 0.869) |
| concept shifting | (0.122/ 0.727) | (0.554/ 0.458) | (0.181/ 0.67) |
| reasoning | **(7.177/ 0.008)** | (0.47/ 0.493) | (1.526/ 0.217) |
| verbal fluency | (0.03/ 0.863) | (1.35/ 0.247) | (0.662/ 0.416) |

Table S6: Relation between age^2^ and CT for the different ROIs of the DMN (SENS + age^2^) with T-values and p-values in brackets.

|  |  | 1000BRAINS | LHAB | Pooled |
| --- | --- | --- | --- | --- |
| left | aDMN | (0.009/ 0.923) | (0.422/ 0.517) | (0.248/ 0.619) |
|  | lateral pDMN | (1.233/ 0.268) | (0.285/ 0.594) | (0.172/ 0.678) |
|  | medial pDMN | (1.927/ 0.166) | (1.63/ 0.203) | (0.001/ 0.976) |
| right | aDMN | (0.498/ 0.481) | (0.124/ 0.725) | (0.534/ 0.465) |
|  | lateral pDMN | (2.8/ 0.096) | (0.032/ 0.858) | (0.943/ 0.332) |
|  | medial pDMN | (1.37/ 0.243) | (0.003/ 0.954) | (0.68/ 0.41) |

Table S7: Quality Control (QC) Measurements of structural brain images (CNR) and surface reconstruction (Euler No.), both calculated using FreeSurfer. Independent-Sample T-Tests revealed significant differences in both, data quality as well as surface reconstruction quality, with the LHAB sample being superior in terms of both.

|  |  | 1000BRAINS | LHAB | T-Test |
| --- | --- | --- | --- | --- |
| Euler No. | lh | -75.21 | -30.93 | **-12.716 (<.001)** |
|  | rh | -68.88 | -30.19 | **-12.262 (<.001)** |
| CNR: gray/white | lh | 1.78 | 1.93 | **-11.793 (<.001)** |
|  | rh | 1.70 | 1.90 | **-15.316 (<.001)** |
| CNR: gray/csf | lh | 1.19 | 1.31 | **-5.973 (<.001)** |
|  | rh | 1.18 | 1.29 | **-5.987 (<.001)** |

Table S8: Relation between age and CT for the different parts of the DMN, when including QC measurements (left and right hemispheric: Euler NO, CNR for gray/white interface, CNR for gray matter/CSF) as covariate into the statistical model (SENS + QC measurements). Age-related differences in CT remain stable, i.e. no significant age-related differences for the anterior parts of the DMN, while all posterior parts show significant age-related decreases in CT.

|  |  |  | 1000BRAINS | LHAB | POOLED |
| --- | --- | --- | --- | --- | --- |
| AGE  (corrected for gender, education, mental & physical wellbeing, euler no (lh,rh), cnr:gray/white (lh,rh), cnr:gray/csf (lh,rh) | Left | aDMN | .350 (.555/.002) | .184 (.668/.001) | .023 (.880/ <.001) |
|  |  | Lateral pDMN | **6.181 (.014/ .042)** | **4.665 (.032/.021)** | **11.738 (.001/.026)** |
|  |  | Medial pDMN | **13.840 (<.001/.060)** | **11.585 (.001/.051)** | **28.392 (<.001/.026)** |
|  | Right | aDMN | .949 (.331/ .004) | .430 (.513/.002) | 1.506 (.220/.003) |
|  |  | Lateral pDMN | **10.140 (.002/.045)** | **5.951 (.016/.027)** | **17.026 (<.001/.037)** |
|  |  | Medial pDMN | **9.689 (.002/.043)** | **14.172 (<.001/.062)** | **28.482 (<.001/.061)** |
